# Single-Cell RNA Analysis of Murine Osteosarcoma Uncovers *Skp2* Function in Metastasis, Genomic Instability, and Immune Activation and Reveals Additional Target Pathways

**DOI:** 10.1158/2767-9764.CRC-25-0294

**Published:** 2026-04-23

**Authors:** Alexander Ferrena, Ranxin Zhang, Jichuan Wang, Xiang Yu Zheng, Barlas Göker, Giulia Barotti, Hasibagan Borjihan, Sung-Suk Chae, Yungtai Lo, Hongling Zhao, Edward L. Schwartz, Rachel Offenbacher, David M. Loeb, Rui Yang, Simone Sidoli, David Geller, Deyou Zheng, Bang Hoang

**Affiliations:** 1 Institute for Clinical and Translational Research, Albert Einstein College of Medicine, Bronx, New York.; 2 Department of Genetics, Albert Einstein College of Medicine, Bronx, New York.; 3 Department of Orthopedic Surgery, Montefiore Medical Center, Albert Einstein College of Medicine, Bronx, New York.; 4 Musculoskeletal Tumor Center, Beijing Key Laboratory for Musculoskeletal Tumors, Peking University People’s Hospital, Beijing, China.; 5 Department of Biochemistry, Albert Einstein College of Medicine, Bronx, New York.; 6 Department of Epidemiology and Population Health, Albert Einstein College of Medicine, Bronx, New York.; 7 Department of Developmental and Molecular Biology, Albert Einstein College of Medicine, Bronx, New York.; 8 Department of Oncology, Albert Einstein College of Medicine, Bronx, New York.; 9 Department of Medicine, Albert Einstein College of Medicine, Bronx, New York.; 10 Department of Molecular Pharmacology, Albert Einstein College of Medicine, Bronx, New York.; 11 Department of Pediatrics, Albert Einstein College of Medicine, Bronx, New York.; 12 Department of Neurology, Albert Einstein College of Medicine, Bronx, New York.; 13 Department of Neuroscience, Albert Einstein College of Medicine, Bronx, New York.; 14 Data Science Institute, Albert Einstein College of Medicine, Bronx, New York.

## Abstract

Osteosarcoma is the most common primary pediatric bone malignancy. One promising new target is *SKP2*, encoding a substrate recognition factor of the SCF E3 ubiquitin ligase that targets p27 for proteasomal degradation, driving cellular proliferation. Knockout (KO) of *Skp2* in an immunocompetent transgenic mouse model of osteosarcoma improved survival, drove apoptosis, and induced antitumor immunity. In this study, we applied single-cell RNA-sequencing (scRNA-seq) to primary osteosarcoma tumors from Osx-Cre conditional *Rb1/Trp53* KO mice. We further compared with models of *Skp2* disruption: *Skp2* KO or disruption of the Skp2–p27 interaction (resulting in p27 overexpression). We report that murine osteosarcoma models recapitulate the tumor heterogeneity and microenvironment complexity observed in patient tumors. *Skp2* disruption led to reduction of T-cell exhaustion and upregulation of interferon (IFN) signaling, as well as induction of cell type–specific replicative and endoplasmic reticulum stress, which we validated with proteomics analysis. Furthermore, we showed that IFN induction was correlated with improved survival in patients with osteosarcoma. Additionally, our scRNA-seq analysis uncovered decreased expression of metastasis-related gene signatures in *Skp2*-disrupted osteosarcoma, which we validated by a strong reduction in lung metastasis in the *Skp2* KO mice. Finally, we report several mechanisms potentially used by osteosarcoma to escape from *Skp2* targeting, including upregulation of *Myc* targets, induction of genomic instability, overexpression of alternative E3 ligases, and lineage plasticity. These mechanistic insights into osteosarcoma tumor biology and *Skp2* function suggest novel targets for new, synergistic therapies, whereas the data and our comprehensive analysis may serve as a public resource for further big data–driven osteosarcoma research.

**Significance:**

Our single-cell study of murine osteosarcoma models uncovers *Skp2* function in metastasis, genomic instability, and immune activation and reveals additional target pathways to overcome resistance to Skp2 disruptions.

## Introduction

Osteosarcoma is a primary bone malignancy defined by spindle cell morphology and abnormal deposition of bone matrix (1, 2). Osteosarcoma is the most frequent pediatric bone cancer with annual US incidence of 3.1 cases per million (3). Disease progression usually involves metastasis to the lungs (4). For cases that are nonmetastatic at diagnosis, 5-year survival is 70%, whereas for metastatic cases at presentation, comprising 15% to 25% of incidence, the rate is 30% (4, 5). Standard-of-care treatment for osteosarcoma involves cytotoxic chemotherapy and resection, a regimen unchanged since 1986 (6). Genetically, osteosarcoma is complex and involves many DNA copy-number alterations, but the two most frequent mutations are loss of the tumor-suppressors *TP53* and *RB1* (7–9). Histologically, several osteosarcoma subtypes have been described, including osteoblastic osteosarcoma, chondroblastic osteosarcoma, and fibroblastic osteosarcoma, but the molecular distinctions among them and functional implications are unknown, and moreover, clinical management and outcomes are similar between these subtypes (10).

S phase kinase-associated protein 2 (*SKP2*) codes for a substrate recognition factor of the Skp1-Cullin1-F-box (SCF) E3 ubiquitin ligase complex. One key target for ubiquitination and proteasome degradation by the SCF^SKP2^ complex is the cyclin-dependent kinase inhibitor p27, allowing G1–S phase transition and cell-cycle progression (11, 12). *Skp2* knockout (KO) was shown to be synthetic lethal in mouse models of pituitary and prostate cancers in the context of *Rb1* and *Trp53* loss, permanently blocking tumorigenesis in a p27-mediated manner (13). Mechanistically, *Skp2* deletion resulted in hyperactivation of the pleiotropic transcription factor *E2f1*, resulting in both increasing signatures of proliferation but also, critically, in malignant cell apoptosis (14). In transgenic mouse models of osteosarcoma driven by conditional ablation of *Rb1* and *Trp53* in the bone-forming Osx (*Sp7*) lineage, we showed that genetic and pharmacologic inhibition of *Skp2*’s interaction with p27 improved survival, decreased tumor size, and reduced stemness; however, although delayed, tumorigenesis and disease progression still occurred, suggesting a resistance or escape mechanism (15, 16). Furthermore, we showed that KO of *Skp2* resulted in even greater survival improvement and tumor apoptosis in a manner related to *E2f1* (17). Surprisingly, in addition to *E2f1* activity, we also detected increased inflammation in the form of immune infiltration and interferon (IFN) expression in *Skp2* KO osteosarcoma tumors, with the increases associated with improved prognosis in patients with osteosarcoma, suggesting a role of *Skp2* in immunosuppression in osteosarcoma (18). However, in a distinct cancer context, *Myc*-driven Burkitt lymphomagenesis, *Skp2* KO had only a modest improvement in survival (19). The mechanism used by osteosarcoma to escape from *Skp2* disruption, along with the regulatory roles of *Skp2* in the tumor microenvironment, remains unknown.

As previously reported, conditional ablation of *Rb1* and *Trp53* from the bone-forming Osx (Sp7) lineage (“double KO,” “DKO”: Osx-Cre; Rb1^lox/lox^; p53^lox/lox^) forms tumors that strongly resemble osteosarcoma patient tumor histology and disease progression (15). We compared this *Skp2*-intact baseline osteosarcoma model against two models of *Skp2* genetic modulation. The first involved disruption of *Skp2*’s interaction with p27 via a mutation preventing p27 phosphorylation and *Skp2* binding, resulting in a “p27-high” phenotype (“DKO alanine–alanine,” “DKOAA”: Osx-Cre; Rb1^lox/lox^; p53^lox/lox^; p27^T871A/T187A^). The second involved a germline *Skp2* KO (“triple KO,” “TKO”: Osx-Cre; Rb1^lox/lox^; p53^lox/lox^; Skp2^−/−^). In the DKOAA and TKO models, we observed improved survival, delayed tumorigenesis, reduced tumor size, increased tumor inflammation, and induction of apoptosis, relative to DKO (16–18).

Together these previous studies suggest that SKP2 disruption may have multifactorial roles, resulting in delayed but not blocked tumor formation eventually. How the different roles are executed and balanced in tumors can be addressed better using single-cell transcriptomics [single-cell RNA sequencing (scRNA-seq)] analysis to resolve cellular heterogeneity. Therefore, we applied scRNA-seq to compare the transcriptomes of the three transgenic murine osteosarcoma tumor models. With these new data, we first investigated the mechanisms underlying the positive prompts of SKP2 disruption, including reduced metastasis, enhanced antitumor microenvironment, and improved survival. We then studied the potential mechanisms used by osteosarcoma to overcome *Skp2* function loss. With the first reported scRNA-seq dataset of an animal model in osteosarcoma, we also compared it with the scRNA-seq datasets from patient osteosarcoma and validated that the murine osteosarcoma models faithfully recapitulate the complex microenvironment and transcriptomic diversity of human osteosarcoma. Taken together, our data are both valuable for addressing the therapeutic utility of SKP2 disruption and for advance further osteosarcoma research in general.

## Materials and Methods

### Establishment of animal models

Osx-Cre mice, *Rb1*^*lox*/lox^ mice, *Trp53*^*lox*/lox^ mice, *Skp2*^−/−^, and *p27*^*T187A/*T187A^ mice were described previously (15, 16, 20, 21). All mice used for experiments were on FVB, C57BL6J, and 129Sv hybrid backgrounds. First, Rb1^lox/lox^ mice were crossed with Trp53^lox/lox^ mice to generate Trp53^lox/lox^, Rb1^lox/lox^ mice, which were further crossed with Osx-Cre mice to generate Osx-Cre; Trp53^lox/lox^, Rb1^lox/lox^ mice (DKO). The Skp2^−/−^ mice were crossed with Osx-Cre; Trp53^lox/lox^, Rb1^lox/lox^ mice to generate Osx-Cre; Rb1^lox/lox^; Trp53^lox/lox^; Skp2^−/−^ mice (TKO). The *p27*^*T187A/*T187A^ mice were crossed with Osx-Cre; Trp53^lox/lox^, Rb1^lox/lox^ mice to generate Osx-Cre; Rb1^lox/lox^; Trp53^lox/lox^; *p27*^*T187A/*T187A^ mice (DKOAA). Animals were maintained under a pathogen-free condition in the Albert Einstein College of Medicine animal facility, following animal experimental protocols reviewed and approved by Einstein Animal Care and Use Committee (#20180401), conforming to accepted standards of humane animal care. The tumor diameter was measured using a caliper every 3 days, and the relative tumor volume was calculated by the following formula: (length × width^2^) × 0.526. Tumors were resected when their volumes reached approximately 500 mm^3^.

### scRNA-seq data generation

Mice of each model presenting tumors close to 1.5 cm in diameter were humanely euthanized. One tumor each from four TKO, three DKOAA, and three DKO animals was carefully dissected, removing surrounding tissues, and then rinsed thoroughly with cold PBS three times to eliminate impurities. Following the standard 10X Genomics sample preparation protocol, the samples were sectioned into 1 to 2 mm pieces and subjected to digestion with type II collagenase and trypsin. To ensure complete digestion, the samples were incubated in a shaker at 37°C and shaken every 10 minutes. After 1.5 hours of digestion and incubation, the cell suspension was filtered through a strainer and centrifuged to remove the enzymes. Subsequently, red blood cell (RBC) lysis buffer was added to the cell suspension to eliminate RBCs. The number of cells was quantified using a Cell Counter (Bio-Rad, #TC-20), and only samples with an appropriate cell density (1,000 cells per μL) and a live-cell percentage greater than 85% were used for further scRNA-seq.

Cells were then loaded into a 10X Chromium instrument (10X Genomics) using the Chromium NextGEM Single Cell 3′ GEM, Library, and Gel Bead Kit v3 at the Einstein Genomics Core. Sequencing of the DNA libraries was performed using an Illumina HiSeq 4000 system (Novogene; scRNA-seq at E9.5 and E10.5) with 150  bp read length.

### scRNA-seq data analysis

#### Preprocessing and alignment

Sequencing reads in FASTQ format were demultiplexed and processed using CellRanger (v6.0.1; 10X Genomics, RRID: SCR_017344) and aligned to the mm10 *Mus musculus* reference genome (mm10-2020-A; preprepared by 10X Genomics).

#### Filtering of RBCs, low-quality cells, and doublets

We devised several methods for filtering of poor quality cells, which are described in detail at https://github.com/FerrenaAlexander/FerrenaSCRNAseq. In brief, we applied all filtering steps to each sample individually. For filtering of RBCs and other low complexity cells, defined as cells with very few unique genes detected given the number of unique molecular identifiers (UMIs), we devised a two-step regression approach, in which a linear regression and LOESS regression model were fit with log(number of UMIs) as the predictor and log(number of unique genes) as the dependent variable. Barcodes with LOESS residuals < −4 and linear model Cook’s distance >4/Ncells were called as low-complexity outliers and discarded. Next, we selected cells with cutoffs of >1,000 nUMIs, >200 unique genes, <25% mitochondrial UMIs, and <25% hemoglobin-related UMIs. We next used data-driven filtration based on median absolute deviation: an upper cutoff of median + median absolution deviation × 2.5 for cutoffs of mitochondrial content, and a lower cutoff of median – median absolute deviation × 2.5 for nUMIs. The final cutoffs for each of the filtering steps were described in Supplementary Table S1. Then, using Seurat (v4.4.0, RRID: SCR_016341), we clustered each sample using the sctransform workflow, using 3,000 features (default for scTransform), 30 principal components (PC), and Louvain resolution of 0.1 and all other default parameters (22, 23). Finally, using these clusters, we applied DoubletFinder (v2.0.2) with option “sct” = T, and other parameters were kept as default to exclude doublets (24).

#### Integration, clustering, and cell marker analysis

For integration and batch correction of scRNA-seq samples, RISC (v1.6) was used (25). Sample DKO_1 (DJ582M11) was used as the reference sample, PCs 1:30 were used for integration and batch correction, and 10 neighbors were used for Louvain clustering within RISC, whereas all other parameters were default. Then, Seurat (v5.0.2) was used for marker analysis via the FindAllMarker function with the only.pos parameter set to true. For marker prioritization, a marker score was calculated, consisting of [(pct.1 – pct.2) × acg_log_2_ fold change], which emphasizes specificity in marker gene expression.

#### Mutation calling from scRNA-seq analysis and malignant cell annotation

For calling single-nucleotide variations and clustering based on cell mutational data, Souporcell (RRID: SCR_027462) was used (26). In most samples, clear distinction of malignant versus stromal clusters was possible using k = 2. For some samples in which the distinction was unclear, k = 3 was used.

For calling copy-number variations (CNV), InferCNV (RRID: SCR_021140) was used (https://github.com/broadinstitute/inferCNV). Macrophages were used as nonmutant reference cells, because these cells were present in all samples. Raw (non–batch-corrected) counts were supplied to InferCNV. CNV calling was done separately for nonmalignant stromal cells and malignant cells, as InferCNV was unable to run on the entire dataset. A mouse reference annotation GTF file (GenCode VM23) was supplied to InferCNV. For malignant cell analysis, the InferCNV “analysis_mode” parameter was set to “subclusters” to capture intratumor heterogeneity. Hidden Markov model (HMM) gene-level CNV results were used from InferCNV for downstream analysis (InferCNV output filename: “20_HMM_pred.repr_intensitiesHMMi6.leiden.hmm_mode-subclusters.Pnorm_0.5.infercnv_obj”). Malignant and stromal results were read in and harmonized, with macrophage CNV calls excluded from downstream analysis. To study the numbers of genes affected by CNVs for each cell, the number of genes with HMM status not equal to 1 was counted. To study the number of 2× deletions or amplifications per cell, the number of genes with HMM status equal to 0 and greater than or equal to 2 was counted. The HMM matrix was also used for PC analysis (PCA) for all cells and for malignant cells only, using the top 1,000 most highly variable features for both.

**Figure 1.**
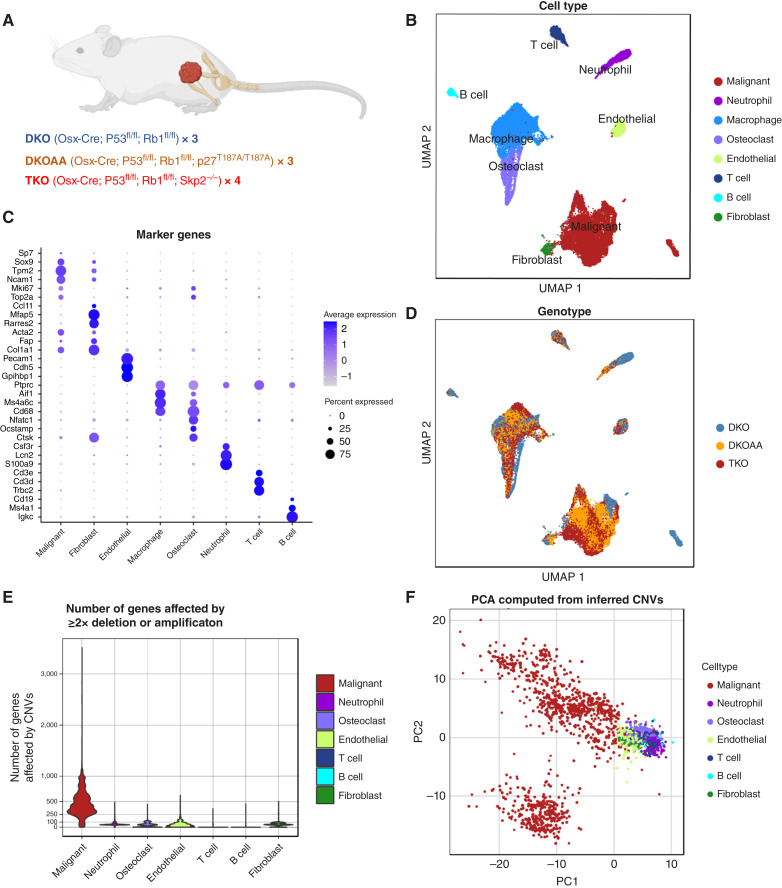
Complex microenvironment in transgenic murine osteosarcoma tumors. **A,** Mouse models for scRNA-seq analysis. Numbers indicate the mice included, with one tumor isolated from each mouse for scRNA-seq. **B,** UMAP of integrated cells colored by cell types. **C,** Canonical and data-driven markers of cell types shown as a bubble plot. **D,** UMAP colored by genotype. **E,** Violin plot of number of genes affected by “extreme CNVs” (deep 2× deletions, or 2×> amplification) in individual cell types. **F,** PCA plot of all cells using their CNV profiles inferred from scRNA-seq data. UMAP, Uniform Manifold Approximation and Projection.

Clusters were annotated to cell types based on marker analysis and low number of CNVs for immune cells and endothelial cells, using the markers shown in Fig. 1. Final cell annotations for malignant cells and cancer-associated fibroblasts were based on marker analysis, Souporcell clustering, and InferCNV results. The fibroblast cluster was annotated as such because its small number of inferred CNVs matched that of immune and endothelial cells and clustered with the rest of the microenvironment in Souporcell results for most samples. Integrated clustering took precedence over individual sample clustering in cell annotation.

### Differential expression analysis across TKO, DKOAA, and DKO

To take advantage of replicates, a pseudobulk based strategy was used to reduce false positives (27). For each cell type, UMIs for all cells were summed up by sample for each gene, and then gene expression among the three osteosarcoma models were compared via the EdgeR (RRID: SCR_012802) likelihood ratio test (28). Gene set enrichment analysis (GSEA) was applied via the FGSEA package for pathway analysis (https://www.biorxiv.org/content/10.1101/060012v3; ref. 29). Gene sets, including the Hallmarks gene sets, were derived from the Molecular Signatures Database via the “Msigdbr” package (30, 31). For network analysis of enriched pathways, aPEAR (v1.0.0 from CRAN) was used (32).

### Subclustering and differential compositional abundance analysis

For subclustering of individual cell types, RISC batch-corrected data from each cell type was subsetted, and Seurat (v4.4.0) was used to identify the top 2,000 most highly variable genes and scale the expression of these genes before PCA. For most cell types, PCs 1:30 and Louvain resolution of 0.5 was used. In some cell types, poor overlap between samples was observed, and marker analysis did not reveal clear cluster markers, so these values were adjusted. In particular, for subclustering of malignant cells, PCs 1:10 and resolution 0.1 were used, whereas for macrophages, PCs 1:30 and resolution 0.7 were used. Markers were derived for each subcluster using Seurat function FindAllMarkers with the parameter “only.pos” set to true.

Compositional analysis was also applied in a pseudobulk-aware manner to reduce false positives (33). For compositional analysis between the three osteosarcoma models, the propeller test was used with option “transform” set to “asin” for arcsine square root transformation, as recommended by benchmarking analysis (bioRxiv 2022.02.04.479123; ref. 34).

### Differential CNV analysis across TKO, DKOAA, and DKO

To quantify the increased heterogeneity among CNV profiles of TKO and DKOAA samples in a replicate-aware manner, we used comparisons of Euclidean distance in the PC space. We first computed replicate centroids in PC space by taking the mean PC values from each replicate’s cells. Next, we computed group centroids for DKO, DKOAA, and TKO by taking the mean replicate centroid values per group. We then compared the replicate-to-group centroid Euclidean distances between TKO, DKOAA, and DKO using two-sample *t* tests.

To compare patterns of CNVs across the three osteosarcoma models, we devised two novel replicate-aware methods. Both were based on the gene-level HMM from InferCNV (see above). In the first, which we referred to as the “differential CNVs mean” or “DiffAmps_mean” approach, we took the mean CNV state of all cells for each sample, resulting in a matrix of CNV gene by sample, in which values were the mean CNV state of all cells from that sample. Then, for each gene, the differences between osteosarcoma models were compared in a replicate-aware manner via Wilcoxon test. In the second method, which we referred to as the “differential CNVs count” or “DiffAmps_count” approach, we separately considered amplifications and deletions. For each gene, the number of cells with HMM state of 0 (2× deletion) or 2 or more (2× or higher amplification) was counted. Then, for each sample, the proportion of cells with or without 2× deletion or amplification was calculated. For each sample, this produced a matrix of gene by sample, in which each value was the proportion of cells with extreme (2×) CNV. Because this was equivalent to compositional abundance analysis, we then transformed the proportions using the arcsine square root transformation and compared via *t* test, similar to propeller analysis (see above). For both methods, *P* < 0.1 is used, on the basis of this being a secondary, exploratory analysis. However, to increase biological relevance, only genes that were also significantly overexpressed by differential expression analysis were considered for downstream analysis and literature search.

### Signature score analysis, cell-cycle analysis, and Myc proportion analysis

To study the expression of groups of genes, the previously described module score method was used to compute signature score for the gene group (35). This method is implemented in Seurat in the “AddModuleScore” function. To study cell-cycle phase, we used the related “CellCycleScoring” with genes derived also from Seurat lists of S phase and G2/M phase genes. To study *Myc* responder cells, we used all genes from the “Hallmark *Myc* targets V1” gene set to calculate *Myc* target signature scores among malignant osteosarcoma cells. Next, we defined *Myc*-responder–high cells as those with a score above the median value determined for all malignant cells in all samples. Then, we compared proportions of *Myc*-responder–high cells between osteosarcoma models in a replicate-aware manner using the propeller test with the “asin” transformation, as described see above.

### Human osteosarcoma scRNA-seq data analysis

To compare murine osteosarcoma with human patient osteosarcoma, we downloaded the data from two studies that applied scRNA-seq to human patient osteosarcoma tumors (33, 36). Data were downloaded from the Gene Expression Omnibus (GEO; accessions numbers: GSE152048 and GSE162454). Datasets were integrated, batch-corrected, and clustered using RISC (v1.6) as described above for the mouse osteosarcoma data. Cell annotations were not shared publicly by either study, so we performed cluster marker analysis using Seurat (v5.0.2) FindAllMarkers function with only.pos set to true. For immune cells, clusters were annotated using marker genes. To identify malignant osteosarcoma cells and nonmalignant, nonimmune stromal cells, InferCNV was applied, using macrophages as reference cells. InferCNV was applied to each sample individually, and then HMM results were harmonized across samples. Macrophage CNV calls were not used in downstream analysis. Malignant clusters were called based on the presence of CNVs and markers, whereas fibroblasts and pericytes were called based on their lack of CNVs and markers.

To study the fidelity of murine osteosarcoma to human tumors, markers from murine cell types were used. Using the package “biomaRt” (v2.54.1, RRID: SCR_019214; https://feb2021.archive.ensembl.org/; ref. 37), human orthologs were found, and only markers with a single unambiguous ortholog were retained. Using these ortholog lists, signature scores for each murine cell type were calculated in the human osteosarcoma data. The average score for each human cell type was calculated, and then a correlation matrix was computed and used for hierarchical clustering.

### NCI TARGET osteosarcoma survival analysis

We downloaded clinical and transcriptomic data from the NCI TARGET osteosarcoma cohort using the TARGET data portal (38). After downloading the data, we filtered for samples which we could harmonize with clinical and survival data and removed samples with abnormally low TPM values, leaving 83 patients. The R package biomaRt was used to find orthologous pairings between mouse and human genes, whereas genes that could not be unambiguously mapped were removed (37). To calculate expression signature scores for the murine osteosarcoma marker lists, the TKO and DKOAA upregulated IFN scores, and the TKO upregulated myogenesis score, we used the module score method (35). For survival analysis with the module scores, we used the survival (v3.4.0) and survminer (v0.4.9) packages in R for Kaplan–Meier (KM) analysis. For KM, we dichotomized the module scores at the median value to compare survival in high versus low with KM and log-rank tests.

### Label transfer from bone atlas and inference of osteosarcoma pathologic subtype

To infer pathologic subtypes (osteoblastic, chondroblastic, and fibroblastic), a scRNA-seq dataset of nonmalignant murine bone cells was used (39). Counts and metadata were downloaded from the Broad Institute single-cell portal (accession: SCP361). Cell annotations were simplified to combine cell clusters together by cell type (i.e., fibroblastic 1, 2, 3, 4, and 5 were combined to “fibroblastic”). Data were downloaded and processed with the Seurat “sctransform” function. Then, Seurat label transfer was used. For each murine osteosarcoma sample, malignant cells were selected, and then the function FindTransferAnchors was used with healthy bone as the reference and malignant osteosarcoma as the query, PCs 1:30, and normalization method set to “SCT.” Then, TransferData was used to acquire inferred cell types and scores. To call a whole tumor as a pathogenic subtype, the transferred cell type with the highest proportion among malignant cells was used as the label. To compare proportions of microenvironment cell types between osteoblastic and fibroblastic subtypes, in which multiple samples were detected, we used the propeller test.

### Metastatic status evaluation of lung

Mice of each model with observable limb tumors that met euthanasia criteria were continuously included in the study. After euthanasia, whole lung tissue was collected from 19 DKO mice, 17 DKOAA mice, and 38 TKO mice. The harvested lung tissue was subsequently rinsed in cold PBS and fixed with formalin. Paraffin blocks were created, and serial sectioning was performed at consistent intervals, with 10 to 30 intervals per lung determined based on lung size. A total of 480 intervals from DKO mice, 400 from DKOAA mice, and 515 from TKO mice were obtained. Each interval underwent hematoxylin and eosin (H&E) staining and was examined for metastatic lesions by two practicing clinicians. The proportion of lungs and intervals affected by metastatic lesions was calculated and compared among the various groups via Fisher exact test.

### Establishment of TKO, DKOAA, and DKO cell lines

TKO, DKOAA, and DKO cell lines were established previously (16, 17). Briefly, primary cells were extracted from mouse osteosarcoma tumor tissues once the tumors reached a diameter of approximately 1.5 cm. The harvested tissues were minced and dissociated using collagenase II in Eagle Minimum Essential Medium for 30 to 60 minutes at 37°C. The dissociated cells were filtered through a 70-μm cell strainer to isolate single cells, which were then expanded, passaged, and stored in liquid nitrogen. The identity of the isolated cells was monitored via morphologic assessment under light microscopy, which remained consistent with characteristic osteosarcoma cell morphology throughout the culture period. The cultures showed no visual signs of microbial contamination, and no specific *Mycoplasma* testing was performed. For proteomic analysis, the frozen cells were thawed and cultured for recovery. All analyses were conducted using cells within 10 passages of initial isolation to maintain phenotypic stability. Approximately 1 to 1.5 million cells were plated 1 day before harvest. Cells were harvested by rinsing twice with PBS, scraping, and pelleting by centrifugation. The cell pellets were stored at −80°C until processing.

### S-trap protein digestion for proteomics

TKO, DKOAA, and DKO cell lines were homogenized with a probe sonicator in a buffer containing 5% SDS, 5 mmol/L DTT and 50 mmol/L ammonium bicarbonate (pH = 8) and left on the bench for about 1 hour for disulfide bond reduction. Samples were then alkylated with 20 mmol/L iodoacetamide in the dark for 30 minutes. Afterward, phosphoric acid was added to the sample at a final concentration of 1.2%. Samples were diluted in six volumes of binding buffer (90% methanol and 10 mmol/L ammonium bicarbonate, pH 8.0). After gentle mixing, the protein solution was loaded to an S-trap filter (Protifi) and spun at 500 g for 30 seconds. The sample was washed twice with binding buffer. Finally, 1 µg of sequencing grade trypsin (Promega), diluted in 50 mmol/L ammonium bicarbonate, was added into the S-trap filter, and samples were digested at 37°C for 18 hours. Peptides were eluted in three steps: (i) 40 µL of 50 mmol/L ammonium bicarbonate, (ii) 40 µL of 0.1% trifluoroacetic acid (TFA) and (iii) 40 µL of 60% acetonitrile and 0.1% TFA. The peptide solution was pooled, spun at 1,000 g for 30 seconds and dried in a vacuum centrifuge.

### Sample desalting preparation for mass spectrometry

Prior to mass spectrometry (MS) analysis, samples were desalted using a 96-well plate filter (Orochem) packed with 1 mg of Oasis HLB C-18 resin (Waters). Briefly, the samples were resuspended in 100 µL of 0.1% TFA and loaded onto the HLB resin, which was previously equilibrated using 100 µL of the same buffer. After washing with 100 µL of 0.1% TFA, the samples were eluted with a buffer containing 70 µL of 60% acetonitrile and 0.1% TFA and then dried in a vacuum centrifuge.

### LC-MS/MS acquisition and analysis

Samples were resuspended in 10 µL of 0.1% TFA and loaded onto a Dionex RSLC Ultimate 300 (Thermo Fisher Scientific), coupled online with an Orbitrap Fusion Lumos (Thermo Fisher Scientific). Chromatographic separation was performed with a two-column system, consisting of a C-18 trap cartridge (300 µm ID, 5 mm length) and a PicoFrit analytic column (75 µm ID, 25 cm length) packed in-house with reversed-phase Repro-Sil Pur C18-AQ 3 µm resin. Peptides were separated using a 90 minutes gradient from 4% to 30% buffer B (buffer A: 0.1% formic acid, buffer B: 80% acetonitrile + 0.1% formic acid) at a flow rate of 300 nL/minute. The mass spectrometer was set to acquire spectra in a data-dependent acquisition (DDA) mode. Briefly, the full MS scan was set to 300 to 1,200 m/z in the Orbitrap with a resolution of 120,000 (at 200 m/z) and an AGC target of 5 × 10^5^. MS/MS was performed in the ion trap using the top speed mode (2 seconds), an AGC target of 1 × 10^4^, and an HCD collision energy of 35.

### Proteomic data analysis

Proteome raw files were searched using Proteome Discoverer software (v2.5, Thermo Fisher Scientific) using SEQUEST search engine and the SwissProt mouse database (updated April 2023). The search for total proteome included variable modification of N-terminal acetylation and fixed modification of carbamidomethyl cysteine. Trypsin was specified as the digestive enzyme with up to two missed cleavages allowed. Mass tolerance was set to 10:00 pm for precursor ions and 0.2 Da for product ions. Peptide and protein false discovery rate was set to 1%. Following the search, data were processed as described (40). Briefly, proteins were log_2_-transformed, normalized by the average value of each sample, and missing values were imputed using a normal distribution two standard deviations lower than the mean. Next, PCA was performed, revealing one sample (“DKO_1”) to be an outlier (Supplementary Fig. S9A). Cross-sample comparisons found that this sample had relatively fewer protein numbers detected and its protein abundance was also trended to low; thus, it was removed from subsequent analysis. Protein expression levels were compared across models via two-sample *t* test in R (v4.3.2).

## Results

### A complex tumor microenvironment is present in the transgenic murine osteosarcoma tumors

Ten tumors obtained from our three mouse models of osteosarcoma (4 TKO, 3 DKOAA, and 3 DKO) were submitted for scRNA-seq using the 10X Genomics platform (Fig. 1A; Supplementary Table S1). After strict quality control of the data, cells from all samples were integrated, clustered, and annotated. In all samples, we detected malignant cells along with diverse immune cells (macrophages, osteoclasts, T cells, B cells, and neutrophils) and nonmalignant stromal cells (endothelial cells and cancer-associated fibroblasts; Fig. 1B; Supplementary Fig. S1). This assortment of cell types closely matches the observations in two recent scRNA-seq studies of human patient osteosarcoma tumors (33, 36). Cell types were annotated by the top markers computed from the data (i.e., data-derived markers) and additionally canonical markers for each cell type (Fig. 1C). Interestingly, malignant cells only expressed osteoblast lineage factor Osterix (*Sp7*) sparsely and were also positive for chondroblastic lineage factor *Sox9*, indicating that lineage infidelity may be a feature of advanced osteosarcoma tumors. Although all the cell types were present in all models, transcriptomic distinctions in some cell types existed even after batch correction and data integration, such as malignant cells, indicating *Skp2*’s roles in osteosarcoma development (Fig. 1D).

Whereas most of the cell types could be annotated by their distinct transcriptomic profiles and marker genes (Fig. 1C; Supplementary Fig. S1; Supplementary Tables S2–S4), the classification of malignant osteosarcoma cells versus nonmalignant mesenchymal stromal cells (MSC), especially fibroblasts, was less clear, likely reflecting the mesenchymal cell origin of osteosarcoma. To resolve this, we called sequence mutations from the scRNA-seq data, considering that the malignant cells should have more mutations compared with their nonmalignant counterparts. We started with single-nucleotide variations and small indels using Souporcell, which clusters cells by their mutation profiles (26). For most samples, such as DKO_1, DKOAA_1, and TKO_1, the analysis revealed a clear difference between suspected malignant osteosarcoma cell clusters and nonmalignant stromal cells (Supplementary Fig. S2). For some tumor samples, such as DKOAA_2, a complex multi-clonal malignant population seemed to be present, supported by two transcriptomically distinct clusters. For other samples, such as DKO_2, the separation of malignant and nonmalignant cell types was more ambiguous and only possible after examining the integration coclustering with cells from all samples.

OS tumors display high levels of genomic instability and high frequency of large-scale CNVs (41). We next turned to CNV analysis with the expectation that malignant cells would have more CNVs than stromal cells. For this, we used inferCNV, which has been shown to identify CNVs reliably from scRNA-seq data at a level matching to the results called from whole-exome sequencing of paired synovial sarcoma tumors (42). Indeed, our inferCNV analysis detected clear differences between malignant and nonmalignant cells, especially when considering the data for (2×) deletions of both gene copies or 2× or more amplifications (referred as “extreme CNVs”; Fig. 1E; Supplementary Fig. S3). To further support our identification of malignant cells, we performed PCA using the inferred CNV profiles. The results showed high complexity and diversity of CNVs among malignant cells, separating them from nonmalignant cells that had significantly fewer CNVs regardless of cell type (Fig. 1F).

In short, this comprehensive, integrative cell annotation approach enables us to reliably distinguish malignant cell clusters and all other cell types, thus allowing us to proceed with the downstream analysis with greater confidence. Additionally, this analysis revealed that *Rb1* and *Trp53* ablation in the Osterix (*Sp7*) lineage produces tumors that strongly resemble human osteosarcoma in terms of microenvironment cell makeup and genetic instability (further discussion below).

### *Skp2* KO reduces molecular signatures of invasiveness and incidence of lung metastasis in OS

In patients with osteosarcoma, disease progression typically manifests as aggressive metastasis to the lung, and if unresponsive to salvage treatment, results in death (4). We previously reported that TKO and DKOAA mice both display significantly improved survival relative to DKO (17). Additionally, knockdown of *SKP2* expression or pharmacologic *SKP2* inhibition in xenograft tumors derived from patient osteosarcoma cell lines led to reduced metastasis in SCID mice (43).

**Figure 2.**
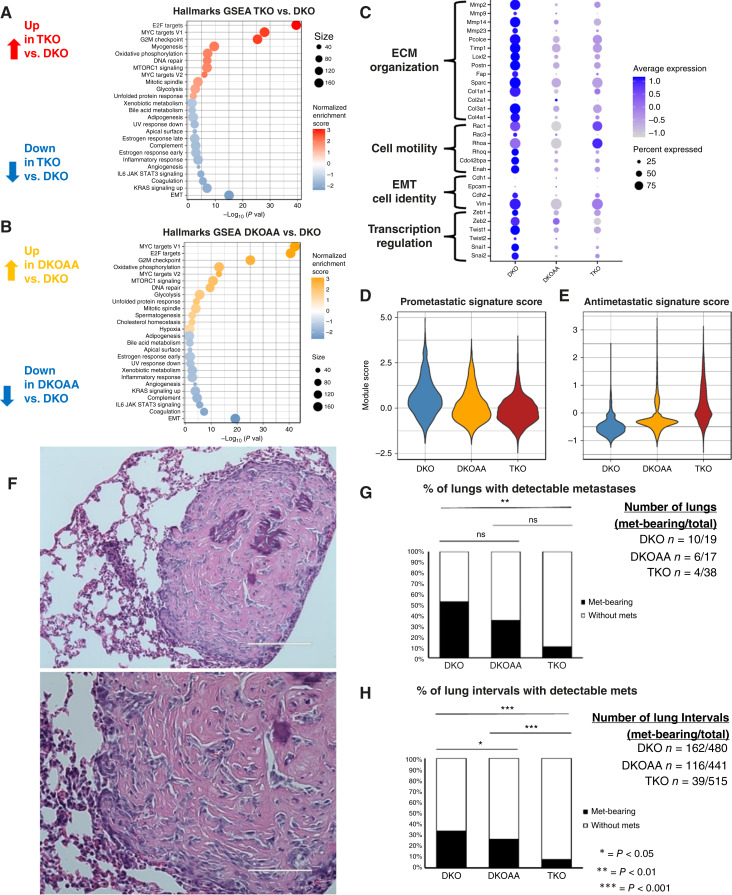
*SKP2* KO reducing osteosarcoma lung metastasis and genes related to invasiveness. **A,** Dotplot of Hallmarks gene sets significantly more active in TKO relative to DKO in malignant cells. **B,** Dotplot of Hallmarks gene sets significantly more active in DKOAA relative to DKO in malignant cells. **C,** Dotplot showing aggregated single-cell expression differences among OS models for genes in EMT-related phenotypes, including ECM organization, cell motility, cell identity, and transcriptional regulation. **D** and **E,** Signature gene signatures scores of prometastatic and antimetastatic OS genes (44). **F,** Representative H&E image of lung metastasis tumor from TKO at 200 μm (top) and 100 μm (bottom) scales. **G,** Quantification and comparison of tumor-bearing lungs. **H,** Quantification and comparison of tumor-bearing serially sectioned lung intervals.

To study the effect of *Skp2* functional disruption on osteosarcoma malignant cells, we performed differential expression analysis between TKO, DKOAA, and DKO cells and uncovered many pathways significantly enriched among the differentially expressed genes (DEG; Fig. 2A and B; Supplementary Tables S5 and S6). Among the downregulated pathways in both TKO and DKOAA malignant cells, epithelial–mesenchymal transition (EMT) stands out [Fig. 2A and B (bottom)].

EMT has been linked to metastasis in many cancers, including osteosarcoma, and involves dramatic functional and cellular morphologic changes. These include gain of cell motility and capacity for modification of the extracellular matrix (ECM), key features related to local invasiveness and distal metastasis (45). Many genes related to ECM organization and cell motility along with related transcription factors were downregulated in TKO (Fig. 2C). However, markers of epithelial cell identity were not strongly expressed in any tumor, indicating that the difference is driven by functional phenotypes and that malignant osteosarcoma cells did not activate the epithelial programs. Accordingly, we observed significant downregulation of gene sets related to ECM organization and cell motility in both TKO and DKOAA relative to DKO malignant cells (Supplementary Fig. S4).

This expression difference in EMT-related genes suggests that TKO and DKOAA tumors may have reduced capacity for invasiveness and metastasis relative to DKO. To further investigate this, we used recently reported gene signatures to calculate prometastasis and antimetastasis signature scores (46). We found the highest prometastasis score in DKO tumors, an intermediate score in DKOAA, and the lowest prometastasis score in TKO (Fig. 2D). Conversely, we observed the opposite trends for the antimetastasis scores (all *P* < 0.01, Wilcoxon test; Fig. 2E).

We next directly tested the incidence of lung metastasis in the three osteosarcoma models. Using analysis of H&E-stained formalin-fixed, paraffin-embedded lung samples, we were able to easily distinguish osteosarcoma lung metastases from normal lung tissue (Fig. 2F). Quantification of H&E images from lungs revealed that the percent of lung metastases was significantly reduced in TKO compared with DKO (*P* < 0.05; Fisher exact test, Fig. 2G). DKOAA tumors had a lower but nonsignificant reduction in metastasis-bearing lungs. To better quantify metastasis extent within each lung, we also analyzed serially sectioned lung intervals and detected a significant reduction of metastasis-bearing lung intervals in both TKO and DKOAA relative to DKO, as well as a significant reduction in TKO relative to DKOAA (Fig. 2H).

Taken together, our scRNA-seq analysis and *in vivo* study both indicate that *Skp2* KO reduces the incidence of lung metastasis in osteosarcoma. This suggests that *Skp2* disruption in osteosarcoma has antitumor benefit by downregulation of EMT and metastasis-related gene expression.

### Functional disruption of SKP2 induces immune activation in the form of increased IFN pathway activity and reduction of T-cell exhaustion

Our prior work on *SKP2* in osteosarcoma revealed increased inflammation in TKO relative to DKO tumors, alongside evidence of increased infiltration of macrophages, T cells, B cells, and endothelial cells, as well as upregulation of IFNγ response (18). Additionally, we showed that TKO and DKOAA mice have improved survival compared with DKO, with tumors from these models showing increased tumor apoptosis and reduced stemness compared with DKO tumors (16, 17). We sought to expand on these findings, which were from bulk tumors, using scRNA-seq data to determine whether the effects are cell type–specific.

**Figure 3.**
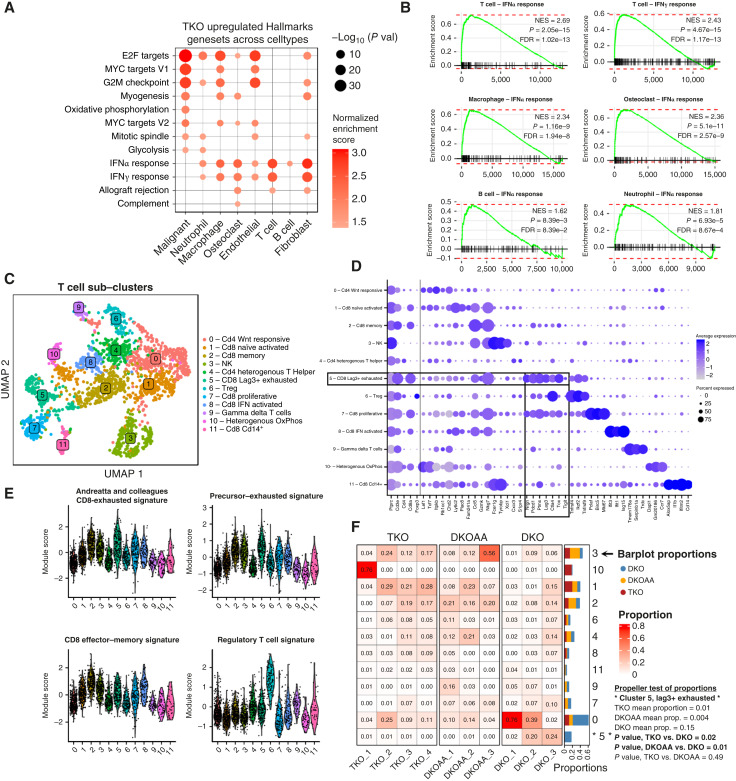
Induced immune activation in the form of IFN pathway activity and reduction of T-cell exhaustion. **A,** Dotplot of Hallmarks gene sets significantly upregulated in TKO relative to DKO across cell types. **B,** GSEA plots showing enrichment of IFN response pathways in genes differentially expressed in TKO relative to DKO among immune cells. **C,** UMAP showing subclustering of T cells. **D,** Canonical and data-driven markers of T-cell subclusters. **E,** Signature gene scores of T-cell states derived from marker genes in a published meta-analysis of tumor-infiltrating T cells (47). **F,** Compositional analysis of T-cell subclusters across 3 osteosarcoma tumors. UMAP, Uniform Manifold Approximation and Projection.

We thus performed differential gene expression analysis across TKO, DKOAA, and DKO and identified DEGs that were significantly enriched in many pathways in individual cell types (Fig. 3A; Supplementary Figs. S5 and S6). Notably, in all microenvironment cell clusters, IFN response pathways were significantly upregulated in TKO relative to DKO, with the strongest upregulation of both type 1 and type 2 IFN response observed in T cells (Fig. 3B). In DKOAA, IFN response pathways were also upregulated, but significant upregulation was restricted to T cells and osteoclasts (Supplementary Fig. S7A). Upregulated IFN response genes were numerous and included transcription factors like *Irf5* and *Irf7*; antiviral genes such as *Oas2*; stress response regulatory genes such as *Eif2ak*; antigen presentation genes such as *H2-Q7*; tetratricopeptide-containing IFN-induced genes such as *Ifit3*; and proinflammatory cytokines such as *Cxcl10* (Supplementary Fig. S7). Interestingly, among upregulated genes in the IFN response pathways were E3 ubiquitin ligase genes such as *Trim21*, *Trim25*, and *Trim26*. *Trim21* that have previously been demonstrated to bind with *SKP2*, causing degradation of p27 (48). We also found upregulation of *Stat1* in T cells (Supplementary Table S5).

One potential mechanism underlying the increased immune activity in TKO tumors may be related to cellular stress response, as endoplasmic reticulum (ER) stress and replicative stress have both been linked to IFN signaling (49, 50). We therefore checked for differential expression of pathways related to stress and unfolded protein response (Supplementary Fig. S8). Surprisingly, we detected significant upregulation of unfolded protein response in malignant cells and endothelial cells in TKO and DKOAA. However, TKO macrophages seemed to downregulate this pathway, whereas DKOAA macrophages showed upregulation of the pathway. These findings underscore the value of scRNA-seq analysis, uncovering cell type–specific effects that could be missed in the analysis of bulk tumors. Interestingly, we also observed upregulation of gene sets related to replicative stress (including genes such as *Clspn* and *Chek1*) in malignant cells, macrophages, and endothelial cells, as well as telomere stress (with genes such as *Acd*) in malignant cells. These findings suggest a link between the hyper-replication phenotype observed in *Skp2* KO and inflammation via induction of cellular stress in malignant cells and macrophages. Given these findings, further exploration of the mechanistic relationship between *SKP2*, ER stress, and replicative stress is warranted.

To validate our findings in protein levels, we performed proteomic analysis and found that many of these genes also showed an expression change at the protein level. Using cell lines established from primary tumors of each model, we found a strong global correlation between protein abundance in our cell line models and RNA expression of the malignant cells in our scRNA-seq data, and we detected many differentially expressed proteins (Supplementary Fig. S9; Supplementary Table S7). Some of the protein changes are consistent with our prior studies (16, 17). Notably, we observed increased levels of proteins involved in antigen presentation, including MHC class 1 genes such as *H2-K1*, as well as upregulation of genes associated with stress granules such as *G3bp1*. Stress granules are membrane-free aggregations of proteins and RNA which form in conditions of cell stress (51). We note that genes, including *Skp2*, *Myc*, and the E2F transcription factors, were not detected in our MS data, suggesting the importance of the tumor microenvironment during tumorigenesis.

We next investigated genes related to IFN signaling (Supplementary Fig. S10). We found that the expression of IFN ligands in our scRNA-seq data was sparse and restricted to specific cell types, whereas upstream regulators *Tmem173* (STING), *Cgas*, and *Tbk1*, as well as IFN receptors, were more uniformly detected. Nevertheless, we did observe evidence of modest upregulation of upstream pathway genes *Tmem173* among B cells and *Cgas* among endothelial cells, receptor gene *Ifnlr1* among neutrophils, and type 1 IFN ligand *Ifna4* among macrophages in TKO relative to DKO (*P* < 0.05, EdgeR). We also performed cell–cell communication analysis using CellChat with our scRNA-seq data (52). IFN signaling was not detected by CellChat, likely due to low levels of the ligand expression, but it did detect a multitude of significant signaling differences across the three models (Supplementary Fig. S11). Considering the complexity in different cell types and how they can cross-talk, more research will be required to study the mechanism underlying IFN pathway induction in the osteosarcoma tumors.

To investigate how each cell type was affected by *SKP2* disruption in a higher resolution, we carried out subclustering analysis using batch-corrected data (see “Materials and Methods”). Various transcriptomically distinct cellular subpopulations were discovered, and their corresponding markers identified (Supplementary Figs. S12 and S13; Supplementary Table S8). For tumor-infiltrating T cells, subclustering distinguished 11 T-cell subtypes and additionally a separate cluster expressing NK markers (Fig. 3C). As in the full dataset with all cells, we annotated these subclusters based on canonical markers in the literature and data-derived top marker genes (Fig. 3D). Notably, this uncovered a CD8 T-cell subcluster (cluster 5) characterized by high expression of dysfunctional T-cell “exhaustion” markers, such as PD-1 (*Pdcd1*), *Ctla4*, *Tigit*, and especially *Lag3*, along with exhaustion regulator *Tox*. To corroborate our results, we obtained gene signatures from a prior meta-analysis (53) of scRNA-seq datasets of tumor-infiltrating T cells and used them to compute signature scores for different T-cell states, including a CD8 exhaustion signature, a closely related “precursor-exhausted” signature, a CD8 effector–memory signature, and a regulatory T-cell signature (Fig. 3E). Concordant with our marker-based annotation, the results showed that the T-cell subcluster 5, relative to all other subclusters, displayed a stronger expression of the “CD8-exhausted” (*P* < 0.001, Wilcox test) and “precursor-exhausted” (*P* < 0.001, Wilcox test) signature genes. Although these exhaustion signatures were also high in subcluster 2, the top signature for that subcluster was “CD8 effector–memory” (*P* < 0.001).

Using this T-cell subcluster annotation, we compared their abundances between DKO, DKOAA, and TKO tumors (Fig. 3F). Interestingly, the only significant difference was a reduction in the proportion of subcluster 5, the CD8 *Lag3*+ exhausted T cells, in both TKO and DKOAA compared with DKO. Although CD8 exhausted T cells accounted for a large proportion of tumor-infiltrating T cells in DKO tumors, they were almost completely absent from TKO and DKOAA tumors.

Several other cell types also exhibited significant subtype alterations among DKO, DKOAA, and TKO (Supplementary Fig. S14; Supplementary Table S9). These included *Naalad*2+ malignant subcluster 4, significantly depleted from both TKO and DKOAA tumors relative to DKO; *Camp+*/*Fcnb−* neutrophil subcluster 2, enriched in DKOAA relative to DKO; *C1qa+* osteoclast subcluster 0, enriched in DKOAA relative to DKO; *Chil3+* osteoclast subcluster 4, depleted from DKOAA relative to DKO; *Kcne3*+ endothelial cluster 6, enriched in DKOAA relative to both TKO and DKO; *Hs3t1*+ B cell cluster 2, depleted in TKO relative to DKO; and *Ighd*+ B cell cluster 1, enriched in DKOAA relative to DKO. Although these results suggest a strong cell population difference in the osteosarcoma tumors with disrupted *Skp2* function, the functional importance and clinical relevance of our observations will require more validation and further study.

Taken together, our results indicate that immune activation in osteosarcoma, in the form of induction of IFN activity and downregulation of T-cell exhaustion, is likely a key consequence of *SKP2* function disruption. The mechanism underlying immune activation may be related to induction of cellular stress in TKO and DKOAA malignant or microenvironment cells. Additionally, the concordance of many of these results between the TKO and DKOAA osteosarcoma tumors indicates that the mechanism underlying this immune activation involves the function of p27.

### E2f activity was upregulated in TKO and DKOAA malignant cells

**Figure 4.**
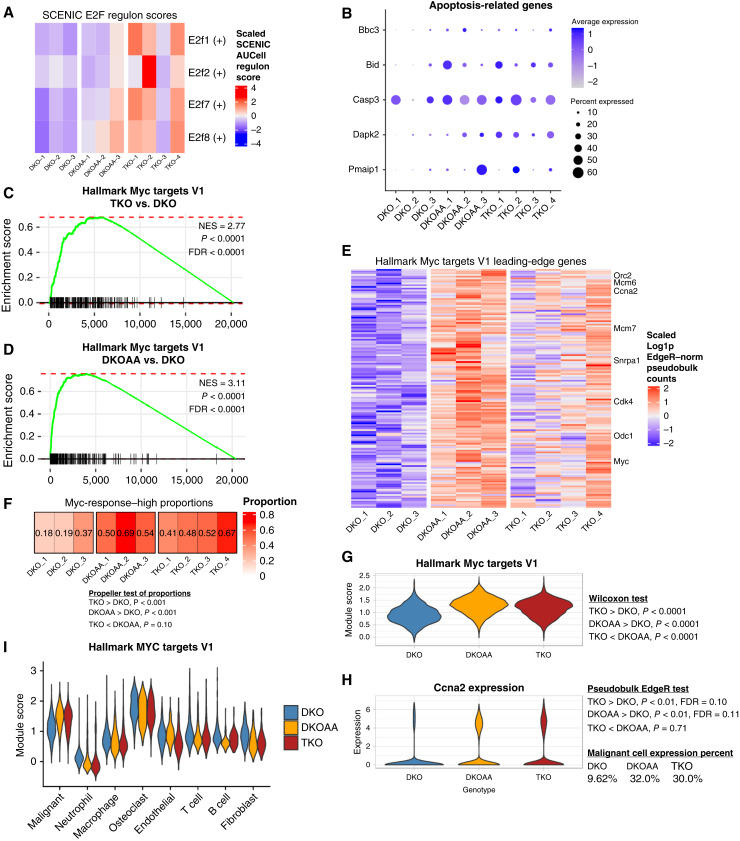
Upregulated Myc target activity in TKO and DKOAA malignant cells. **A,** SCENIC transcription factor activity scores of E2F family transcription factors. **B,** Expression of apoptosis-related genes derived from the Reactome apoptosis gene set. **C** and **D,** GSEA plot of one of the “Hallmark Myc targets V1” gene set in TKO and DKOAA relative to DKO, respectively. **E,** Heatmap of GSEA leading-edge genes in the “Hallmark Myc targets V1” gene set. The intersect of leading-edge genes from the TKO and DKOAA enrichments is shown. **F,** Compositional analysis of *Myc*-high responder cells. **G,** Violin plot for malignant cells only of signature scores calculated from genes in the “Hallmark Myc targets V1” gene set. **H,** Violin plot for malignant cells of *Ccna2*, a *Myc* target gene. **I,** Violin plot of the *Myc* signature scores in all cells.

*Skp2* KO has been shown to effectively prevent tumorigenesis in other cancers driven by p53 and Rb1 inactivation, such as prostate and pituitary cancers, via E2F1-driven apoptosis (13). *Skp2* deletion in this context was synthetic lethal, resulting in hyperactivation of *E2f1*, cell-cycle progression to S phase but a p27-driven inhibition of complete cell division, and a lethal DNA rereplication phenotype that ultimately led to malignant cell apoptosis (13, 14). Our prior work also showed an increase of apoptosis in TKO and DKOAA tumors. Consistent with these reports, E2f target upregulation was observed in TKO and DKOAA malignant cells (Fig. 2A and B). To further investigate this, we applied SCENIC to predict and compare the activity of the E2F family of transcription factors (Fig. 4A; Supplementary Fig. S15). From our scRNA-seq data, we were able to infer SCENIC regulon activity scores for *E2f1*, *E2f2*, *E2f7*, and *E2f8*. Among malignant cells, the regulon scores of *E2f1* (*P* = 0.03), *E2f7* (*P* = 0.02), and *E2f8* (*P* = 0.03) were significantly greater in TKO than in DKO, whereas only *E2f8* was significantly higher in DKOAA than in DKO (*P* = 0.03, *T* test). Nevertheless, numerous target genes of the E2F family showed upregulation in TKO and DKOAA relative to DKO malignant cells (Supplementary Fig. S15G–S15J). Additionally, we investigated cell-cycle phases and uncovered evidence of cell cycling among malignant cells and osteoclasts but to a lesser degree in other cell types (Supplementary Fig. S15D). Comparison of S phase and G2/M phase score distributions revealed an increase in S phase among malignant cells compared with G2/M, whereas both were upregulated in TKO and DKOAA relative to DKO. The differences of these scores were less consistent in other cell types across the three osteosarcoma models (Supplementary Fig. S15E and S15F). Critically, a link between *SKP2* disruption, E2f hyperactivation, and overreplication of DNA was reported to explain the induction of apoptosis upon *Skp2* KO (13). Consistent with this, we found significant upregulation of apoptosis-related genes, including *Bbc3* and *Bid*, in both TKO and DKOAA relative to DKO malignant cells (Fig. 4B; Supplementary Fig. S15L).

Taken together, our scRNA-seq analysis supports the established relationship between *Skp2* functional loss, E2f induction, and apoptosis. The increase of apoptosis and the higher activity of immune microenvironment (described above) may contribute to slow tumor growth and small tumor size in TKO relative to DKO mice (17, 18).

### *Myc* activity was upregulated in TKO and DKOAA malignant cells

Although *Skp2* disruption drives improved survival in murine osteosarcoma, tumors continue to emerge, in contrast to what was observed in other cancer types, suggesting that osteosarcoma uses unique escape mechanisms. One possibility is that osteosarcoma hijacks other elements of the complex regulatory mechanisms controlling cell division to survive *SKP2* disruption. Whereas activation of E2F targets seems advantageous by induction of tumor cell apoptosis, *Myc* targets, which are often considered to drive tumor development, were also significantly upregulated in both TKO and DKOAA malignant cells relative to DKO (Fig. 4C and D). These included metabolism-related genes such as *Odc1*, proliferation-related genes like *Ccna2* and *Cdk4*, and *Myc* (*c-Myc*) itself (Fig. 4E). To further investigate this, we used the “Hallmark Myc targets V1” gene set to compute a signature score and defined cells above the median score as “high–Myc-responder” cells. The proportion of such cells was significantly higher in TKO and DKOAA than in DKO (Fig. 4F). At the single-cell level, the score distribution was greater in TKO and DKOAA than in DKO (Fig. 4G). More TKO and DKOAA cells exhibited strong expression of *Ccna2*, a canonical *Myc* target, than DKO cells as well. However, malignant cells, along with osteoclasts, expressed *Myc* targets at a level relatively higher than most nonmalignant cell types, regardless of model (including DKO), suggesting that *Skp2* functional loss potentiates a *Myc* target transcriptional program that is already activated in the malignant lineage (Fig. 4I; Supplementary Fig. S15M). We also studied *Myc* regulon activity using SCENIC but found only a modest upregulation. This may be due to some difference in the gene sets defined for *Myc* targets between the Hallmark and the SCENIC databases. However, examination of the expression patterns of the *Myc* target genes from SCENIC revealed a similar pattern as those observed in the “Hallmark Myc targets V1” gene set (Supplementary Fig. S14C and S14K). Alternatively, the upregulation of *Myc* targets may be driven by other transcription factors.

Given the lack of impact of *Skp2* KO in *Myc*-driven Burkitt lymphoma (19), our findings suggest that the upregulation in TKO may be a mechanism adopted by osteosarcoma tumors to escape from the lack of *Skp2*. Future studies will be required to address whether there is a direct link between *Skp2* disruption, *Myc* target induction, and persistent (albeit delayed) tumorigenesis in TKO and DKOAA, but *Myc* targets nevertheless represent a potential target for synergistic therapy with *SKP2* inhibition.

### *Skp2* KO places selective pressure on osteosarcoma malignant cells

**Figure 5.**
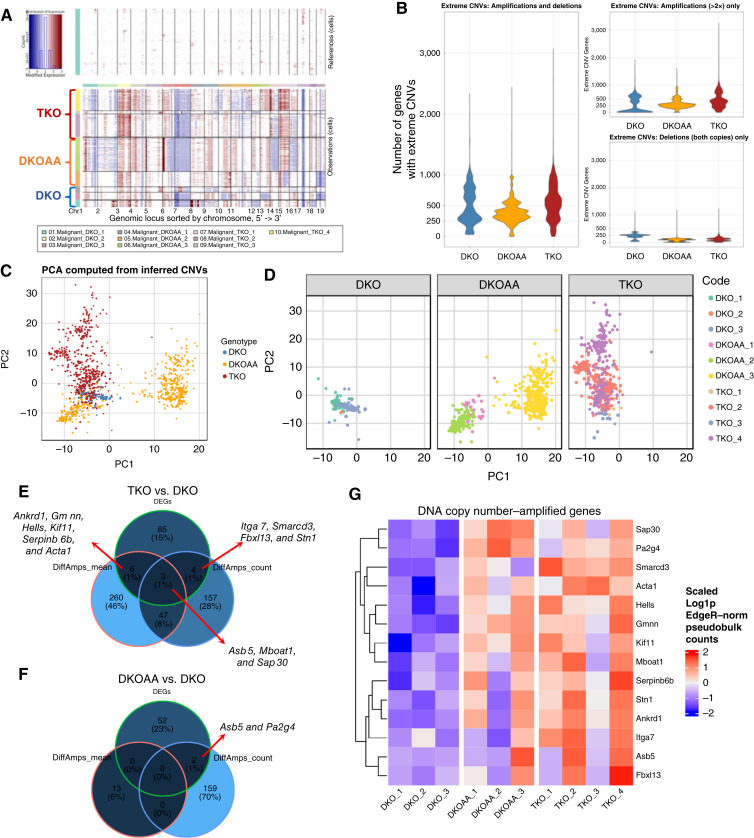
Genome instability in osteosarcoma malignant cells. **A,** Heatmap showing HMM results of CNVs as determined by InferCNV. The top heatmap shows CNV states in macrophages (reference nonmutated cells), whereas the bottom heatmap shows malignant cells from each sample (rows). The columns represent chromosomes. **B,** Violin plots showing the number of genes affected by “extreme CNVs” (2× deletions or 2×> amplification). The left shows all extreme CNVs, the top-right shows extreme amplifications, and the bottom-right shows extreme deletions. **C,** PCA plot of malignant cells using inferred CNVs. **D,** Same PCA split but by OS models and colored by samples. **E,** Venn Diagram showing comparison of two differential CNV identification methods with differential expressed genes of TKO vs. DKO. **F,** Venn diagram showing comparison of two differential CNV identification methods with differential expressed genes of DKOAA vs. DKO. **G,** Heatmap showing expression patterns of significantly DNA copy number–amplified genes that were also significantly overexpressed in TKO and DKOAA.

In addition to *Myc* target activation, we also wanted to explore additional mechanisms that may contribute to escape from *Skp2* functional disruption, leading to tumor formation in the TKO and DKOAA mice. Osteosarcoma is notorious for its complex genomic instability (44, 48), which may provide an evolutionary mechanism by which osteosarcoma tumors resist treatment (54). This may also be related to the frequent *RB1* and *TP53* loss in osteosarcoma. To study whether *Skp2* disruption could further increase genome instability in osteosarcoma, and thus allow tumor development, we inferred CNV profiles from scRNA-seq data and compared them across the three osteosarcoma models. We found large variations among all the tumor samples (Fig. 5A), but some consistent differences among malignant cells of the three osteosarcoma models were observed. The number of genes affected by extreme amplifications (defined as 2× copies or more) was somewhat greater in TKO compared with DKO malignant cells, though this was not a significant shift at the sample level [*P* = 0.6; Fig. 5B (top right)]. To study this at the single-cell level, we performed PCA on the malignant cells, based on what genes were affected by the inferred CNVs. Interestingly, this revealed a greatly increased CNV heterogeneity in TKO and DKOAA than in DKO (Fig. 5C). Further study of the PCA embeddings demonstrated that DKO tumor samples seemed quite similar to one another, whereas TKO and especially DKOAA malignant samples had extremely diverse CNV profiles from DKO and from one another (Fig. 5D). To quantify this in a replicate-aware manner, we performed analysis of the PC embeddings based on replicate-to-genotype centroid distances (see “Materials and Methods”). An increased replicate-to-genotype centroid distance would indicate more heterogeneity among replicates in a given group. We found that replicate-to-group centroid distance was significantly higher in TKO versus DKO (*P* = 0.03, two-sample *t* test) but less so in DKOAA versus DKO (*P* = 0.11). This indicates greater intragroup heterogeneity among the TKO cells versus DKO, consistent with a more complex mutational profile after *Skp2* disruption.

We next investigated whether specific genes affected by CNVs could be identified among DKO, DKOAA, and TKO, despite high within-group variability. We devised two replicate-aware, pseudobulk-based methods for comparison of CNVs across the models, the first involving comparison of mean inferred CNV states, and the second counting up CNVs and comparing proportions (see “Materials and Methods”). Although we did not detect any significantly DEGs, we found some genes significantly more amplified in (multiple) TKO and DKOAA tumors relative to DKO, some of which were also significantly overexpressed in TKO and DKOAA malignant cells (Fig. 5E and F). More such genes were detected from TKO compared with DKOAA, which may be a result of the high variability observed among DKOAA samples.

Among the genes found to be both significantly amplified and overexpressed in TKO and DKOAA relative to DKO (Fig. 5G), two genes were especially striking, *Fbxl13* and *Asb5*, two poorly studied E3 ligase substrate–targeting genes. *Fbxl13* is a member of the same leucine-rich F-box family as *Skp2* (which is also known as *Fbxl1*) and also functions as a substrate-recognizing component of the SCF complex. It has been shown to target the centrosome protein *CEP192* for degradation, linking this gene to functions including cell motility and mitosis (55). *Asb5* is a poorly studied gene in the ankyrin repeat and SOCS-box containing protein family, many of which have been shown to cooperate with a different E3 ligase complex involving the scaffold protein *Cul5* via the SOCS domain (as opposed to *Cul1* in the SCF^SKP2^ complex; ref. 56). Other notable amplified and overexpressed genes in TKO include *Mboat1*, a negative regulator of ferroptotic cell death (57); *Sap30*, a histone deacetylase associated with worse prognosis in neuroblastoma (47); several genes related to regulation of DNA replication, including *Stn1*, *Hells*, and *Gmnn*; as well as *Itga7* and SWI/SWF (BAF complex) component *Smarcd3*, both of which have been linked to cancer stemness (58, 59). In DKOAA, besides *Asb5*, we observed amplification of the gene *Pa2g4*, which has been shown to cooperate with histone deacetylases to downregulate *E2f1* target activation (60). Additionally, although the ankyrin repeat–containing transcription factor *Ankrd1* has previously been shown to be upregulated by *SKP2* in a manner dependent upon nonproteolytic ubiquitination of YAP and would thus be expected to be downregulated in TKO, we observed both overexpression and amplification of this gene in TKO, potentially indicating context-dependent mechanisms of regulation in osteosarcoma (61). The functional relevance of these genes, and whether there may be target redundancy between *Asb5*, *Fbxl13*, and *Skp2*, warrants further studies.

These results indicate that the mutation spectrum of osteosarcoma tumors is strongly perturbed by *Skp2* KO in a manner particularly driven by copy-number amplifications. Whereas high variability existed among the malignant cell population, evidence for amplification in some cells was detected among several E3 ligase substrate–targeting genes with potentially similar function like *Skp2*, along with other genes related to cancer stemness, DNA replication, and cell death inhibition. These results suggest that *SKP2* functional disruption may place strong selective pressure or establish a genomic context for osteosarcoma to evolve escape mechanisms.

### Mouse models of osteosarcoma predict improved survival of *SKP2*-disrupted IFN response activity

**Figure 6.**
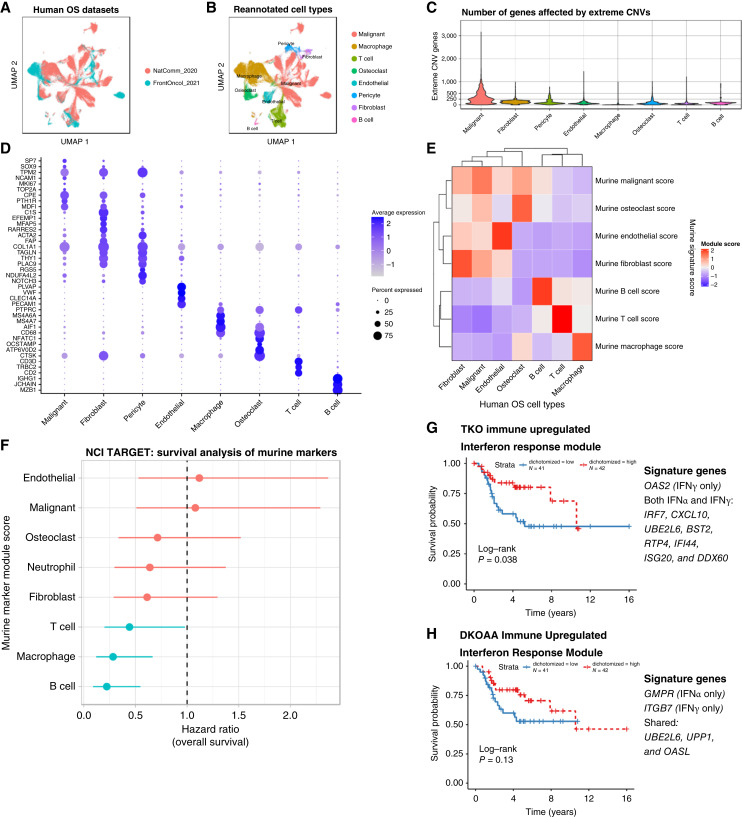
Relating mouse osteosarcoma models to patients. **A** and **B,** UMAPs of human OS patient scRNA-seq data integrated from two publications, colored by data sources (**A**) and cell types (**B**). **C,** Violin plot showing the number of genes affected by extreme CNVs (≥2× deletion or amplification) across cell types. **D,** Canonical and data-driven markers of cell types in integrated human osteosarcoma. **E,** Heatmap showing expression scores of murine OS cell type markers in patient tumor cells. Rows correspond to murine marker genes, and columns refer to human OS cell types. **F,** Forest plot illustrating survival analysis of murine cell type signatures in the NCI Target OS cohort. **G,** KM plot showing survival association of an expression score calculated from genes in the Hallmark IFNα and IFNγ response gene sets that were also upregulated in TKO immune cells relative to DKO. **H,** KM plot showing survival association of an expression score calculated from genes in the Hallmark IFNα and IFNγ response gene sets that were also upregulated in DKOAA immune cells relative to DKO. UMAP, Uniform Manifold Approximation and Projection.

As mentioned above, the cell types present in our immunocompetent autochthonous murine osteosarcoma tumors are similar to those reported from patient tumors (Fig. 1B; refs. 33, 36). To further investigate how well our osteosarcoma models recapitulated human disease, we downloaded and integrated data from 17 human patient tumors (Fig. 6A; refs. 33, 36), which included 11 pretreated, resected samples (“NatComm_2020”) and six treatment-naïve, upfront-resected samples (“FrontOncol_2021”). Cell-level annotations were not provided by either study, so we reannotated cell types after performing cell clustering, marker gene identification, and InferCNV analysis of the integrated data (Fig. 6B; Supplementary Fig. S16; Supplementary Table S10). As in murine tumors, malignant cells from patient tumors have a much higher number of genes affected by 2× deletions or amplifications than stromal or immune cells (Fig. 6C). Additionally, cell type marker genes in human tumors showed expression patterns very similar to those in mouse tumors (Fig. 6D). To better quantify the transcriptomic similarity of cell types between human and mouse osteosarcoma, we identified top marker genes for individual cell types in the mouse osteosarcoma and used them to compute “cell type scores” in the human data. The results indicated high specificity of murine-derived cell type gene signatures (Fig. 6E).

Some cell types, however, were detected in only human or mouse osteosarcoma, including pericytes in human tumors and neutrophils in mouse tumors. Technical factors in sample preparations likely explain this difference. Pericytes may have been insufficiently sampled in murine tumors due to lower sample size (10 mouse tumors vs. 17 human tumors), whereas neutrophils are known to be difficult to capture via scRNA-seq (62), and laboratory conditions may be more suitable for such challenging cells compared with the operating room.

Considering this transcriptomic similarity between murine and patient tumors, we decided to conduct survival analysis in a larger cohort of patients with bulk RNA-seq data, the NCI TARGET cohort, to test the impact of cell types. We observed significant positive survival benefits in the patient samples with greater expression scores for the markers of murine T cells, macrophages, and B cells (Fig. 6F), suggesting that increased abundance of these immune cell types is beneficial to patients. We next studied whether any pathways significantly differentially expressed upon *Skp2* functional disruption were associated with survival. Starting from the Hallmarks IFN (α and γ) response gene sets, we selected the genes upregulated in TKO and DKOAA immune cells to calculate gene scores for the samples in the NCI TARGET cohort. We found that high expression of both were associated with improved survival (Fig. 6G and H).

Taken together, these results indicate that the tumor microenvironment and transcriptome of our murine models of osteosarcoma resemble those of patient tumors and that immune infiltration is associated with improved survival in human osteosarcoma. Furthermore, the IFN response gene signature upregulated in *SKP2*-disrupted osteosarcoma is correlated with improved survival in patients with osteosarcoma.

### Pathologic subtype and lineage infidelity influences osteosarcoma tumor heterogeneity

**Figure 7.**
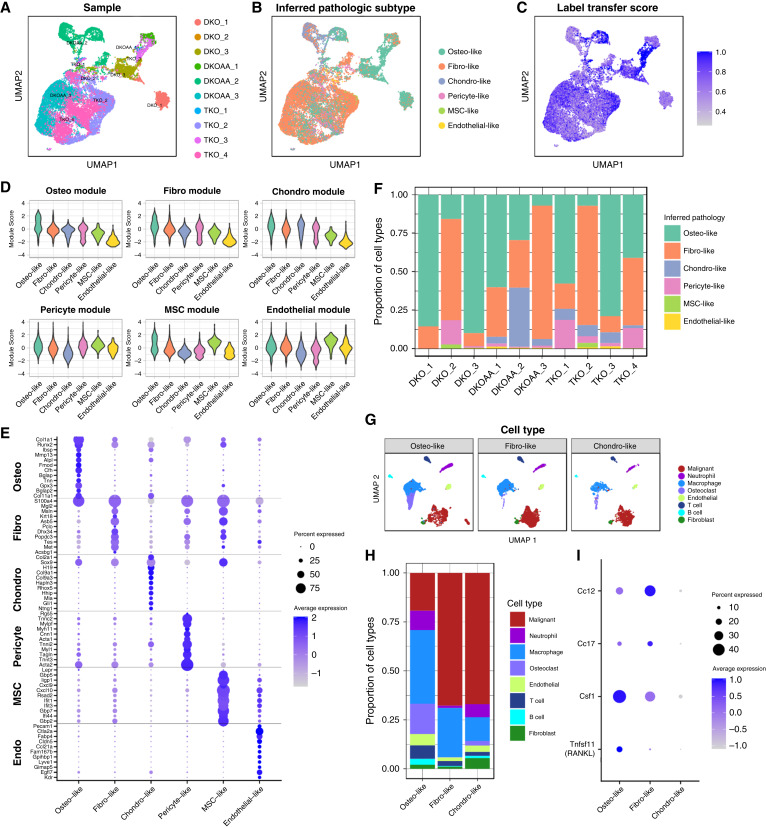
Pathologic subtype and lineage infidelity in mouse osteosarcoma tumor. **A** and **B,** UMAP showing malignant cells from OS tumors, colored by sample (**A**) and pathologic subtype (**B**) as inferred via label transfer from a murine nonmalignant bone atlas dataset. **C,** The same UMAP, colored by label transfer scores. **D,** Violin plots of signature scores computed for markers of various bone cells. **E,** Dot plot of markers of malignant subtype classifications. **F,** Bar plot showing proportions of malignant cells annotated to each pathologic subtype in each sample. The subtype with the highest proportion is considered the inferred pathologic classification of the indicated tumor. **G,** UMAP of all OS tumor cells similar to Fig. 1B but split according to sample-wise inferred pathologic classification. **H,** Proportion of cell types in each pathologic subtype. **I,** Expression patterns of genes related to macrophage and osteoclast differentiation among malignant cells from each subtype. UMAP, Uniform Manifold Approximation and Projection.

Patient osteosarcoma can present in several pathologic subtypes, including osteoblastic osteosarcoma, chondroblastic osteosarcoma characterized by increased chondroid matrix, and fibroblastic osteosarcoma characterized by lower levels of matrix production (63). The first scRNA-seq study of human osteosarcoma reported some clear differences between osteoblastic and chondroblastic osteosarcoma, with the latter characterized by expression of canonical chondroblast markers, including *COL2A1*, *ACAN*, and lineage-driver *Sox9*, reduced presence of osteoclasts in the tumor microenvironment, as well as active differentiation to osteoblastic cell state (36). We wondered whether some levels of tumor heterogeneity in the murine data (Fig. 7A) could be related to pathologic subtypes, although we did not expect *Skp2* KO would shift osteosarcoma subtypes.

To study this, we relied on a published scRNA-seq atlas of nonmalignant bone and bone marrow niche cells (39), because it contained various clusters of osteo-lineage cells (osteoblasts), fibroblasts, chondroblasts, along with pericytes, endothelial cells, and MSCs, which express hematopoietic niche factors such as *CXCL12* and stem cell marker *LEPR*. For the purpose of our analysis, we merged subclusters of the same cell types to obtain clusters related to broad cell types (Supplementary Fig. S17).

We performed a label transfer using Seurat, in which malignant osteosarcoma and the nonmalignant atlas bone cells were integrated and cell labels were estimated based on transcriptomic similarity. The results indicated that osteosarcoma malignant cells were composed mostly of osteo-like, fibro-like, and chondro-like cells (Fig. 7B). Additionally, there were small numbers of cells annotated as pericyte-like, MSC-like, and endothelial-like. Inspection of label transfer score, a measure of transcriptomic similarity between malignant and matching bone atlas cells, revealed high score seemed to correlate with cluster areas with high cell type purity (Fig. 7C).

We also applied label transfer using the same bone atlas to our nonmalignant endothelial and cancer-associated fibroblast cell types (Supplementary Fig. S18). These revealed an almost perfect match for endothelial cells and a high score for most fibroblast cells. Interestingly, some of the fibro-cells were called as osteo-like, which likely explained the lower label transfer scores. This raises the possibility that nonmalignant osteoblast cells may be present in the osteosarcoma tumor microenvironment, alongside (and transcriptomically similar to) cancer-associated fibroblasts.

To further establish the pathologic classifications, we derived markers from the simplified bone atlas dataset for computing signature scores in the malignant osteosarcoma cells (Fig. 7D). This revealed that whereas osteo-like and chondro-like cells strongly express the osteoblast and chondroblast signatures, respectively, the fibro-like cells had only a modest upregulation of the fibroblast signature. The minor cell types, including pericyte-like, MSC-like, and endothelial-like osteosarcoma cells, all had robust upregulation of their respective signature genes. Notably, the osteo-like cells seemed to also express every cell type signature, indicating perhaps more plasticity.

We also inspected the malignant osteosarcoma subtype markers, along with some canonical cell type markers, and observed fairly high expression specificity for most cell types, with the exception of the fibro-like markers, which were expressed somewhat more promiscuously (Fig. 7E). Restriction of marker analysis to the three main subtypes (osteo-like, fibro-like, and chondro-like) revealed more specific markers (Supplementary Fig. S19A). Using SCENIC, we also uncovered numerous transcription factors specific to the main pathologic subtypes (Supplementary Fig. S19B). We noticed that *Asb5* was also found to be overexpressed in the fibroblastic subtype. In concordance with other results, *Asb5* was found to be almost completely silent in nonmalignant subtypes but was upregulated in TKO and DKOAA in all malignant subtypes (Supplementary Fig. S19C and S19D).

Using the inferred pathologic subtypes, we called each tumor sample as either osteo-like, fibro-like, or chondro-like based on which subtypes had the highest frequency in the malignant cells (Fig. 7F). Osteo-like samples included DKO_1 (86% osteo-like cells), DKO_3 (90%), DKOAA_1 (60%), TKO_1 (58%), and TKO_3 (79%); only one sample was classified as chondro-like, DKOAA_2 (38%); and fibro-like samples included DKO_2 (66%), DKOAA_3 (87%), TKO_2 (77%), and TKO_4 (44%). DKOAA_2, the only sample classified as chondro-like, was in fact highly mixed and contained high proportions of all three types (38% chondro-like, 30% osteo-like, 31% fibro-like, and small proportions of others). Although at first we considered this sample as too highly mixed to classify, we decided to keep this result, because the data matched the characteristics of chondroblastic osteosarcoma patient tumors, in which subclusters of intermediate cells transdifferentiating between chondro-like and osteo-like phenotypes were found (36).

Importantly, our osteosarcoma subtype analysis uncovered a clear difference in the makeup of the tumor microenvironment (Fig. 7G). Comparison of the cell type proportions in the microenvironment showed a clear depletion of osteoclast cells in fibroblastic tumors (Fig. 7H). Osteoclasts, macrophage-like hematopoietic cells involved in bone resorption, contributed a large proportion of cells in the osteo-like tumors (16%) but represented a minor proportion in chondro-like tumors (2%) and were exceedingly rare in fibro-like tumors (<0.1%; Osteo vs. fibro *P* = 0.003, propeller test; chondro had just one sample). We noted that osteoclasts exhibited cycling characteristics (Supplementary Fig. S15D), indicative of active osteoclastogenesis in the tumor microenvironment, as in human tumors (36, 64). Based on this, we inspected cytokines involved in this process and observed decreased expression of the macrophage maturation cytokine *Csf1* and osteoclast differentiation factor RANKL (*Tnfsf11*) in the fibro-like and chondro-like malignant cells relative to the osteo-like cells, whereas monocyte homing cytokines such as *Ccl2* and *Ccl7* were not changed (Fig. 7I). This indicates that the rarity of osteoclasts in the chondro-like and especially fibro-like tumors may be a result of reduced tumor-mediated osteoclastogenesis, rather than reduced general monocyte infiltration. Although this demonstrates a clear cell compositional difference among the tumor microenvironment between the osteosarcoma subtypes, the small sample size prevents us from making any clinical or functional implications, but it could be important for future study.

Given these findings, we performed differential expression analysis across osteosarcoma models stratified by pathologic subtype where samples were available, osteo-like and fibro-like. Due to low sample sizes, we were forced to rely on non-pseudobulk methods which may be susceptible to false positives; we applied the Wilcox test. Overall, we found similarity with the nonstratified analysis, including upregulation of *Myc* targets among malignant cells in TKO and DKOAA versus DKO in both osteo-like and fibro-like tumors (Supplementary Fig. S20). However, one notable exception was that EMT-related gene expression was found to be upregulated in TKO and DKOAA osteo-like tumors. Inspection of EMT-related markers indicated that this seemed to be driven particularly by increased expression in TKO of ECM-related genes such as *Mmp23*, *Pcolce*, *Timp1*, *Sparc*, and *Col1a1*, whereas most genes associated with motility remained downregulated in TKO (Supplementary Fig. S21).

Taken together, these results indicate that Osx-cre driven transgenic osteosarcoma can develop to distinct osteosarcoma subtypes as in human osteosarcoma. Our analysis also provides transcriptomic markers of these subtypes that can be evaluated in patient osteosarcoma. Furthermore, the osteosarcoma subtype seems to influence the microenvironment cell composition, e.g., depletion of osteoclasts from chondroblastic tumors as reported in patients and even more pronounced in fibroblastic osteosarcoma. Validations of the inferred subtypes and the corresponding microenvironment difference are needed to confirm our findings; however, our findings indicate that pathologic subtype is a strong source of molecular variation in osteosarcoma tumors that should be considered during sampling in future studies.

### Upregulation of a hazardous myogenic transcriptional program in osteosarcoma with *Skp2* disruption

**Figure 8.**
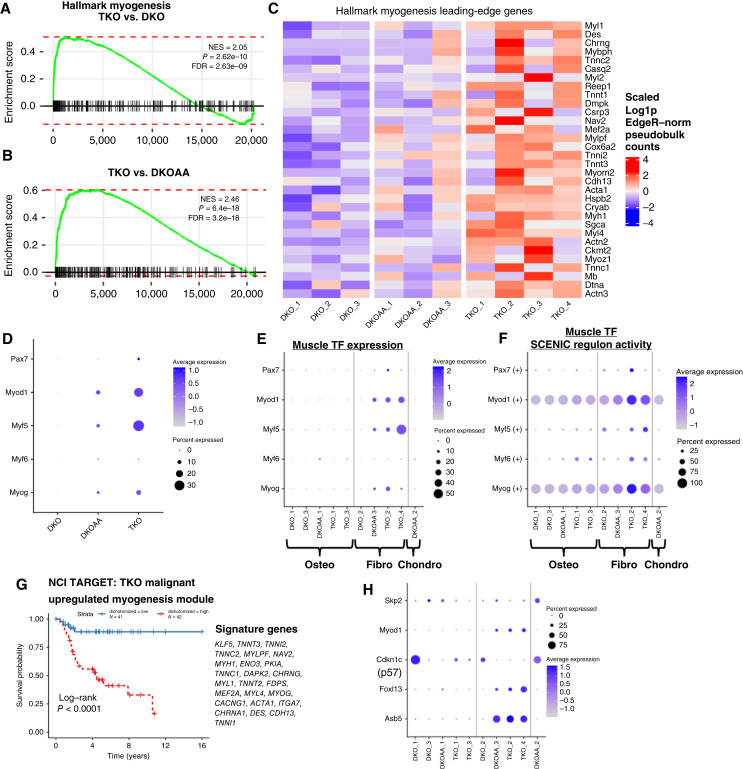
Upregulation of a hazardous myogenic transcriptional program in TKO. **A** and **B,** GSEA plots showing enrichment of the Hallmark myogenesis gene set in TKO vs. DKO and TKO vs. DKOAA, respectively. **C,** Heatmap showing leading-edge genes from the Hallmark myogenesis gene set from GSEA enrichment of TKO vs. DKO. **D,** Dot plot showing expression of canonical muscle transcription factors. **E,** Dot plot showing expression of muscle transcription factors by samples, sorted by inferred pathologic subtypes. **F,** Dot plots showing “regulon activity” as inferred by SCENIC. **G,** KM plot showing survival association of an expression score calculated from genes in the Hallmark myogenesis gene set that were also upregulated in TKO malignant cells relative to DKO. **H,** Dot plot showing expression profiles of key genes related to myogenic signature.

In general, we observed high concordance in the results of comparing TKO and DKOAA to DKO. This indicates that a p27-dependent mechanism is critical for *Skp2* function. However, there are differences between TKO and DKOAA models. For example, TKO malignant cells upregulated genes related to myogenesis relative to DKO, whereas DKOAA did not (Fig. 2A and B). We thus made a direct comparison between TKO and DKOAA and confirmed upregulation of myogenesis genes in TKO relative to DKOAA (Fig. 8A and B). Genes driving this enrichment included transcription factors (e.g., *Mef2a*) and muscle function genes like *Myl1* and *Tnnc2*, components of the sarcomere, an organelle in striated muscle (Fig. 8C).

To confirm this, we examined the expression of transcription factors key for muscle differentiation, myogenic master regulator *Pax7*, and the four myogenic regulatory factors *Myod1*, *Myf5*, *Myf6*, and *Myog*. We found that they were also upregulated in TKO (Fig. 8D and E). SCENIC analysis further confirmed that targets of these transcription factors were more active in TKO (Fig. 8F). Interestingly, there seemed to be a pattern of upregulation in some inferred pathologic subtypes. Fibro-like samples TKO_2 and TKO_4 had the strongest expression and SCENIC regulon scores, followed by DKOAA_2.

We were intrigued by this finding and decided to investigate the clinical relevance of this ectopic myogenesis program. Analyzing the NCI TARGET osteosarcoma cohort, we observed a dismal prognosis in patients highly expressing the TKO myogenesis gene set (Fig. 8G), when the TKO malignant upregulated genes that were also present in the “Hallmark myogenesis” gene set were used to compute myogenesis scores for stratifying the patient cohort. In the osteosarcoma patient scRNA-seq data, most of these genes were specifically expressed by malignant cells but were restricted to one cluster, indicating the program involves a strongly distinct transcriptomic phenotype in osteosarcoma (Supplementary Fig. S22). The human myogenic cluster (malignant C23) was mostly composed of cells from one sample, “NatComm_2022 BC17,” a lung metastatic tumor. Additionally, the expression level of *SKP2* did not seem significantly lower than in other clusters, suggesting that osteosarcoma is capable of myogenic lineage plasticity in a manner that does not involve *SKP2*.

It has recently been reported that *SKP2* was upregulated by *MYOD1* in the context of rhabdomyosarcoma to drive a stem-like state of incomplete, nondifferentiated myogenesis, via degradation of p57, thus linking *Skp2* with myogenesis in sarcoma (65). We did not observe a clear pattern of transcriptional upregulation of p57 (*Cdkn1c*) in TKO or fibroblastic samples, but interestingly, we did detect upregulation of *Asb5* and *Fbxl13* in fibroblastic TKO and DKOAA samples (Fig. 8H). In the human osteosarcoma scRNA-seq data, *ASB5* was also specific to the same malignant cluster that expressed the myogenic signature (Supplementary Fig. S22). *Asb5* expression has been detected in muscle stem cells before (66). It is possible that p57 protein levels may be altered but not reflected at the transcript level and thus still involved in in the myogenic phenotype.

Overall, these results suggest that *Skp2* KO unexpectedly led to upregulation of an abnormal myogenic pathway, perhaps a result of the malignant cells actively exploring evolution landscape of lineage plasticity. As this program was also detected in the human myogenic osteosarcoma cells expressing *SKP2*, it may not be directly regulated by SKP2.

## Discussion

Here, we report the first scRNA-seq study of transgenic murine osteosarcoma, as well as the first report of scRNA-seq in the context of *Skp2* KO. Our findings indicate that the patient osteosarcoma microenvironment is recapitulated by conditional ablation of *Rb1* and *Trp53* in the Osx lineage in mice. Our analysis also uncovered the two sides of targeting *SKP2* functions in osteosarcoma. *Skp2* KO or genetic disruption of its interaction with p27 resulted in increased immune activation, proapoptotic E2f activity, and reduced metastasis-related pathway activities. Using histologic examination of lungs, we validated the latter finding and found a strong reduction in osteosarcoma metastasis after *Skp2* KO. Conversely, our comprehensive analysis of the scRNA-seq data also implicates several potential mechanisms of resistance and escape from the loss of *Skp2*, which may explain why tumorigenesis is not entirely blocked but delayed in the TKO mice. Furthermore, we showed that *Skp2* KO led to increased genome instability in the malignant osteosarcoma cells without *Rb1*/*Trp53*, which may broaden the evolutionary landscape for osteosarcoma to explore. Taken together, our study points to strategies that may be exploited for future development of synergistic *SKP2*-targeted therapies.

We recently established a link between *Skp2* and immunomodulation and reported increased immune infiltration and IFN signaling in *Skp2* KO tumors (18). Specifically, we found that *SKP2* expression was inversely correlated with immune activation in multiple osteosarcoma cohorts as well as in the soft tissue sarcoma–dominated TCGA-SARCOMA cohort and that *Skp2* KO in murine osteosarcoma drove increased immune infiltration into tumors (18). However, those results were obtained from analysis of bulk tumor tissues and thus could not resolve specific cell type effects. Here, we provide new evidence of widespread upregulation of IFN response signaling in multiple cell populations within the osteosarcoma microenvironment upon functional disruption of *Skp2*. Coupled with this, we uncovered evidence of reduced CD8 T-cell exhaustion. Furthermore, we found evidence for increased replication stress and ER stress in several cell types after *Skp2* KO, including malignant cells, macrophages, and endothelial cells. IFN ligand expression was observed to be sparse, but we did detect upregulation of IFN ligands among macrophages. In addition to this, we observed upregulation of activity of the pleiotropic E2f family, including markers of increased cell entry to S phase along with malignant cell apoptosis. Further still, we observed evidence that the complexity of DNA copy-number amplifications was increased in both TKO and DKOAA tumors. Taken together, our data support a model in which *Skp2* KO results in *E2f*-mediated overactivation of proliferative pathways related to DNA replication, resulting in cellular stress and induction of IFN activity (Supplementary Fig. S23). The link between ER stress or replication stress with IFN has increasingly received strong interest as a novel target pathway for immunotherapy in several contexts (49, 50). Our findings support the notion that induction of cellular stress is a viable immunogenic target in osteosarcoma and that *Skp2* disruption induces cell stress in a manner that may be exploited for cytotoxic immune activation. Additionally, it was recently reported that *SKP2* inhibition in orthotopic xenograft models of triple-negative breast cancer led to increased DNA replication and replication stress, resulting in activation of the cGAS/STING pathway and enhancing the efficacy of immune checkpoint blockade therapies (67).

In addition to these, we observed downregulation of metastasis-related signatures in TKO and DKOAA tumors, including downregulation of genes involved in EMT. This seems to be mainly driven by downregulation of genes related to ECM remodeling, cell motility, and associated transcription factors, rather than a shift in “epithelial” or “mesenchymal” cell lineage identity. Consistent with this, we observed a strong decrease in lung metastasis in TKO but only a modest reduction in DKOAA mice. The divergent results between TKO and DKOAA indicate that the mechanism only partially involves the functional interaction between *Skp2* and p27. Alternatively, it is possible that the magnitude of p27 stabilization may differ between TKO and DKOAA, and the differences between models are thus p27 dosage–dependent. Nevertheless, taken together, these data support the use of *Skp2* inhibition for suppressing lung metastasis in osteosarcoma. One interesting future area of investigation would be to study whether *Skp2* KO reduces malignant “redissemination” from established metastatic tumors to novel sites (“metastasis to metastasis” spread). This has received increased interest as a mediator of disease burden in other tumor contexts such as breast cancer (68, 69). Additionally, it would be important to study whether the reduction in metastasis stems from a malignant cell-intrinsic defect, a microenvironment change in the lung or primary tumor stroma, or both. Experimental metastasis models along with rescue experiments may help uncover this in future studies.

Nevertheless, despite the strong survival benefits and delayed tumorigenesis after *Skp2* disruption, osteosarcoma tumors from the TKO and DKOAA mice do eventually grow and progress. Our study points to several potential mechanisms that osteosarcoma tumors may use to escape the functional loss of *Skp2* (Supplementary Fig. S23). Genetically, we detected strong mutational perturbation in TKO and DKOAA relative to DKO tumors, indicating that selective pressure took place, reminiscent of tumor evolution in the context of chemotherapy (54). By analyzing CNV profiles inferred from the scRNA-seq data, we found that numerous genes gained copy numbers in TKO malignant cells, several of which also showed overexpression in TKO and DKOAA, including antiapoptotic genes and understudied E3 ligase genes *Asb5* and *Fbxl13*. Further study, including whether these E3 ligases target substrates similar to those of *Skp2*, or whether these are targetable oncogenes in osteosarcoma or other cancers, is warranted.

Additionally, we uncovered evidence of lineage plasticity among osteosarcoma tumors by upregulation of an abnormal myogenic state. Although we did not have direct evidence for its contribution to osteosarcoma development, interestingly high activation of the myogenic program was associated with poor survival in patients with osteosarcoma. Additionally, lineage plasticity is a mechanism commonly used by cancer to escape treatment (70). We note that whereas we did observe this abnormal myogenic lineage switch in the *Skp2*-disrupted mice tumors, we also detected it in presumably non–SKP2-disrupted human tumor data, specifically, from a human lung metastatic tumor. For this reason, we hypothesize that this apparent promyogenic lineage infidelity may be at least one form of therapeutic resistance from treatment in general in osteosarcoma, rather than just a specific response to SKP2 disruption. This would be consistent with other forms of lineage plasticity reported in other cancers. Nevertheless, this finding warrants further study, for example, to see whether it also occurs when SKP2 is pharmacologically inhibited or whether it may serve as a biomarker for poor response to treatment. Notably, *ASB5* seemed to be correlated with osteosarcoma muscle-related gene expression in both murine and human osteosarcoma and has previously been observed to be expressed in muscle stem cells (66). To our knowledge, this is the first report of malignant myogenic lineage plasticity in osteosarcoma. The mechanism underlying this “osteo-myogenic transition” phenotype, including whether it may be driven by *ASB5*, or whether *ASB5* inhibition may reverse this state, remains an interesting open question. Furthermore, our previous analysis showed adipogenesis also seems to be affected by *Skp2* disruption (16, 18).

In addition to these possibilities, upregulation of *Myc* target activity in TKO and DKOAA tumors relative to DKO seems to be a strong candidate for escape from the loss of *Skp2*. This is further supported by a previous study reporting that *Skp2* KO caused only a modest, nonsignificant improvement in survival in the context of murine models of *Myc*-driven Burkitt lymphoma (19). Our data showed *Myc* upregulation in osteosarcoma tumors but we did not detect amplification of *Myc* or any clear *Myc* targets. In addition, all malignant osteosarcoma cells (including those from DKO) displayed higher *Myc* activity scores, indicating that targeting the *Myc* pathway is likely a good strategy for osteosarcoma treatment and furthermore may increase the efficacy of *SKP2* inhibition. On the other hand, precision medicine approaches, including genomics-informed application of CDK inhibitors, have been proposed in osteosarcoma and were efficacious in *MYC*-amplified patient-derived xenografts but did not completely stop tumor growth (71). Future study of joint *SKP2* and *MYC* inhibition may thus reveal potent synergistic avenues of therapy.

Osteosarcoma is notorious for its extensive heterogeneity across patients. Our study demonstrated that mouse osteosarcoma tumors were also characterized by strong intertumor molecular heterogeneity, as in patients. This heterogeneity may be driven by distinct mutational and CNV profiles as in patients, but we also found transcriptomic and microenvironment differences related to pathologic subtype. It has previously been shown that osteoclastogenesis seems to occur directly in the osteosarcoma tumor microenvironment and that osteoclasts may be rare in the microenvironment of chondroblastic osteosarcoma (36). Consistent with this, we observed evidence for osteoclastogenesis in murine osteosarcoma and reduction of osteoclasts in chondroblastic tumors, but we also found almost complete ablation of osteoclasts from the microenvironment of fibroblastic osteosarcoma, which was correlated with and may be mediated by reduced RANKL signaling. Although the clinical implications of this remain unclear, these data suggest that the variability between osteosarcoma histologic subtypes are strong enough that they should be considered during sampling and statistical power consideration in molecular studies. We also note that there seems to be an overrepresentation of the fibroblastic osteosarcoma subtype among our samples, relative to its rare incidence in humans. It is possible that these tumors may be composed of softer cellular tissue compared with bony matrix found in osteoblastic tumors. The latter require lengthy collagenase treatment, which may reduce cell viability and be considered nonsuitable for sequencing. If that is the case, our finding would represent a sampling bias toward the rare fibroblastic subtype. Alternatively, fibroblastic osteosarcoma may be more common among this model of osteosarcoma than in the patient population. Currently, our sample size is too small to address whether murine models of osteosarcoma driven by Osx-cre ablation of *Trp53* and *Rb1* present with osteosarcoma subtypes at similar proportions to those in human osteosarcoma or whether the proportions are affected by *Skp2* disruption. Future studies could investigate whether alternative methods like single-nuclei RNA-sequencing may achieve better capture of cells from heavily mineralized osteoblastic osteosarcoma tumors. Notably, we did detect upregulation of a promyogenic transcriptomic signature among Skp2-disrupted murine tumors, which seemed to be restricted to the fibroblastic subtype, suggestive of an interaction between SKP2 disruption and pathologic subtype. This may indicate that patient stratification is needed, and SKP2 inhibitors should not be used in the treatment of fibroblastic osteosarcoma. With current standard-of-care osteosarcoma treatments, the pathologic subtypes are treated the same way. This finding indicates that pathologic subtype needs to be accounted for in treatment, or at the very least, in further studies.

A key limitation of our study is the cross-sectional nature of the data, which prevents us from making any inferences about the temporal order of the various events found to be more or less active in the TKO and DKOAA tumors compared with the DKO. Expanding our analysis to samples collected from different osteosarcoma growth stages would allow us to address whether the anticancer pathways (e.g., induction of immune activation) are active predominantly at the early stage, whereas the pro-cancer pathways (e.g., *Myc* target upregulation) are induced at the later stage or concurrently (Supplementary Fig. S23). Knowledge about the order is especially important for targeting SKP2 in patients with osteosarcoma. We should point out that TKO and DKOAA tumors in our study had already successfully developed strategies to cope with *Skp2* disruption partially. Another limitation is that our study was based on transcriptomics and thus may miss posttranscriptional regulatory events. This is important because SKP2 regulates its targets at the protein level and many of the *Myc* and E2F targets are also regulated by protein abundance. We tried to look for this in our proteomic data from OS cell lines, but more studies are needed. Additionally, we should mention that in patients, osteosarcoma tumors are removed and thus may not have the same resistance potentials as discussed for the mouse model. Overall, considering the heterogeneity we observed and in osteosarcoma in general, more samples would be needed to generalize some of our findings.

In conclusion, our study supports the use of *SKP2* inhibition in osteosarcoma treatment but also reveals a complex battery of mechanisms that tumors may exploit for resistance. These suggest that a combination therapeutic strategy using *SKP2* inhibition and other drugs targeting the potential resistance pathways may be promising for osteosarcoma treatment. In clinical practice, it will be important to know which of the potential resistance mechanisms are activated in individual patients. Our study provides biomarkers for molecular mechanisms associated with both effective antitumorigenic *Skp2* inhibition as well as oncogenic resistance pathways.

## Supplementary Material

Supplementary Table S1 Table S1. Sample level metadata.

Supplementary Table S2 Table S2. Markers of clusters. Wilcox test from Seurat (v5) FindAllMarkers function was used.

Supplementary Table S3 Table S3. Markers of celltypes after annotation. Wilcox test from Seurat (v5) FindAllMarkers function was used.

Supplementary Table S4 Table S4. Cell metadata and celltype annotations.

Supplementary Table S5 Table S5. Differential Expression results for all genes, celltypes, and comparisons

Supplementary Table S6 Table S6. GSEA pathway results.

Supplementary Table S7 Table S7. Mass spectrometry differentially expressed protein results.

Supplementary Table S8 Table S8. Marker genes for all celltype subclusters.

Supplementary Table S9 Table S9. Compositional analysis (differential abundance analysis) comparing proportions of cell subtypes from each genotype using the Propeller test from the Speckle package.

Supplementary Table S10 Table S10. Annotations of integrated human OS scRNAseq data from published studies.

Supplementary Figure S1 Figure S1. Clustering and markers of transgenic OS tumors.

Supplementary Figure S2 Figure S2. Souporcell results.

Supplementary Figure S3 Figure S3. side by side comparison of InferCNV results for stromal cells (top) and malignant cells (bottom).

Supplementary Figure S4 Figure S4. Leading edge genes from GSEA showing downregulation of invasive phenotypes in TKO and DKOAA relative to DKO.

Supplementary Figure S5 Figure S5. Differential pathway gene set enrichment analysis among TKO vs DKO in all cell types, visualized as networks via aPEAR.

Supplementary Figure S6 Figure S6. Differential pathway gene set enrichment analysis among DKOAA vs DKO in all cell types, visualized as networks via aPEAR.

Supplementary Figure S7 Figure S7. GSEA of DKOAA and leading edge genes of interferon alpha and gamma response.

Supplementary Figure S8 Figure S8. GSEA of genesets related to cellular stress response.

Supplementary Figure S9 Supplementary Figure S9. Mass spectrometry proteomics comparisons of TKO, DKOAA and DKO malignant tumor cell-derived cell lines.

Supplementary Figure S10 Figure S10. Analysis of Interferon related genes.

Supplementary Figure S11 Figure S11. CellChat analysis of cell signaling differences between OS tumors.

Supplementary Figure S12 Figure S12. Subclustering of individual cell types.

Supplementary Figure S13 Figure S13. Marker analysis for subclusters of individual cell types.

Supplementary Figure S14 Figure S14. Heatmaps showing proportions of subclusters for cell types containing at least one subcluster with a significant difference of proportion among OS models.

Supplementary Figure S15 Figure S15. E2f and Myc upregulation in TKO and DKOAA malignant cells.

Supplementary Figure S16 Figure S16. Clustering and markers of integrated human OS scRNA-seq data

Supplementary Figure S17 Figure S17. Simplification of bone cell atlas annotations for usage in label transfer.

Supplementary Figure S18 Figure S18. Label transfer from bone atlas to non-malignant fibroblasts and endothelial cells.

Supplementary Figure S19 Figure S19. Markers and transcription factors from malignant cells stratified by pathologic subtype.

Supplementary Figure S20 Figure S20. DE across OS models, stratified by pathologic subtype for which samples were available (including Osteo and Fibro-like, but not chondro-like).

Supplementary Figure S21 Figure S21. EMT gene expression in Osteo vs non-Osteo samples.

Supplementary Figure S22 Figure S22. Expression of TKO myogenesis program in human OS tumors.

Supplementary Figure S23 Figure S23. Working model summarizing main findings from mouse OS models.

## Data Availability

The transcriptomic data generated in this study are publicly available in the GEO (accession number: GSE262743). The MS-based proteomics data generated in this study are publicly available in ProteomeXchange (accession: PXD059605). For exploratory data analysis, we provide two web-based Shiny apps, generated using the ShinyCell method (72). For murine data, the app is available at https://scviewer.shinyapps.io/mouseos_scrnaseq_tko_dkoaa_dko/. For integrated human data, the app is available at https://scviewer.shinyapps.io/humanos_scrnaseq/. Analysis code is available at GitHub (https://github.com/bioinfoDZ/OS_scRNA/) and CodeOcean (https://codeocean.com/capsule/5884387/tree/v1).
