# Supplementary material for: Single-Cell RNA Analysis of Murine Osteosarcoma Uncovers Skp2 Function in Metastasis, Genomic Instability, and Immune Activation and Reveals Additional Target Pathways
Source: Cancer Res Commun. 2026 Apr 23;6(4):923–45. doi: 10.1158/2767-9764.CRC-25-0294 (PMC13103941; doi:10.1158/2767-9764.CRC-25-0294)

Supplementary Figure S1: Clustering and markers of transgenic OS tumors. A: Louvain clusters of integrated scRNA-seq data (from RISC). B: Violin plot of number of genes affected by "extreme CNVs" (>=2x deletion or amplification). C: Canonical and data-driven markers plotted for the clusters in A; the same markers as Fig 1C are shown. D: Data-driven markers for each cluster. E: Data-driven markers for each cell type after merging clusters for the same cell type. F: Correlation matrix and hierarchical clustering of cell types across samples, based on average gene expression in each cell type per genotype. G. UMAP of Osx (*Sp7*) expression pattern.

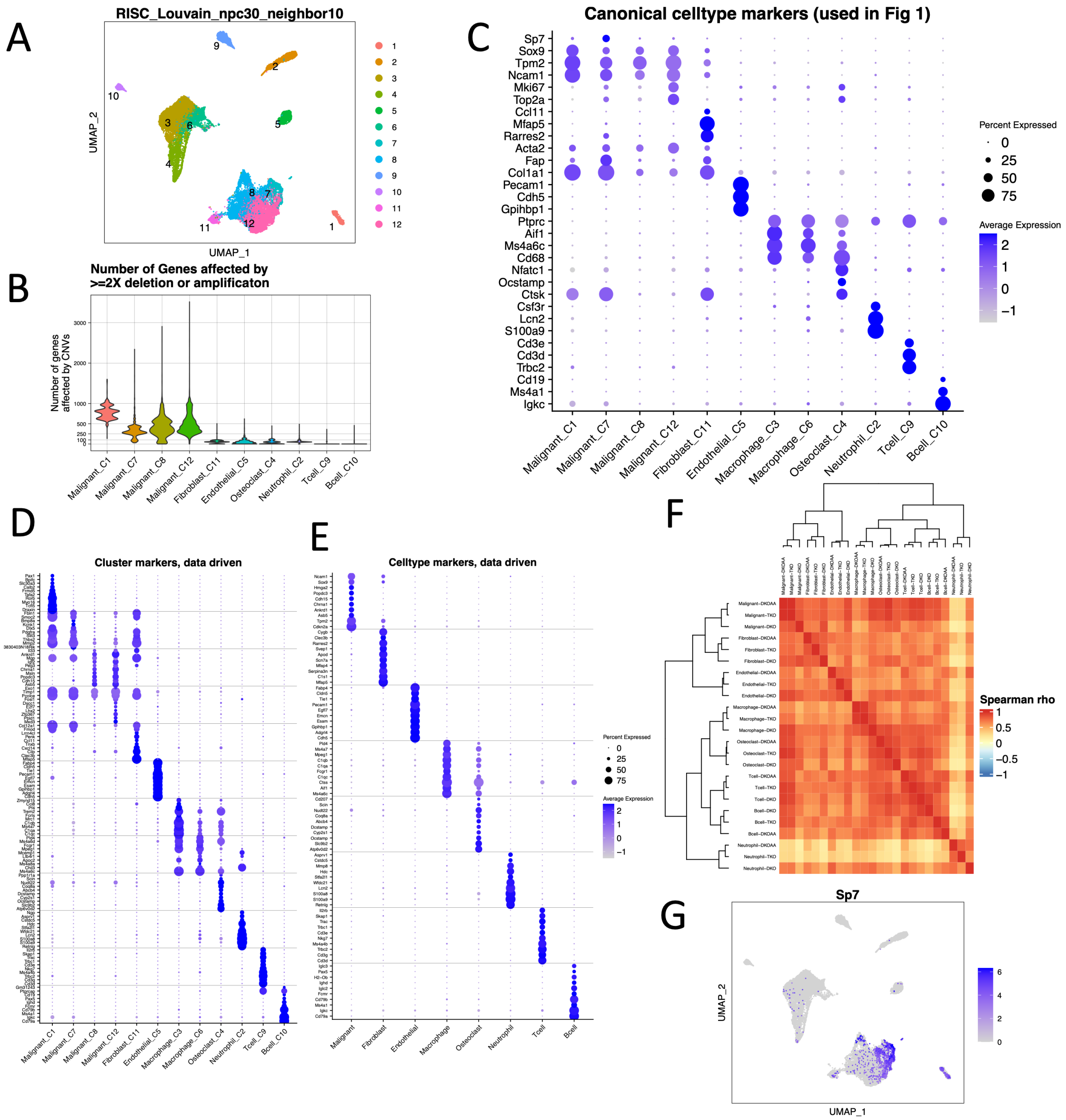

Supplement: Supplementary Figure S1 — Figure S1. Clustering and markers of transgenic OS tumors. [file crc-25-0294_supplementary_figure_s1_suppsf1.pdf]
