# Supplementary material for: Single-Cell RNA Analysis of Murine Osteosarcoma Uncovers Skp2 Function in Metastasis, Genomic Instability, and Immune Activation and Reveals Additional Target Pathways
Source: Cancer Res Commun. 2026 Apr 23;6(4):923–45. doi: 10.1158/2767-9764.CRC-25-0294 (PMC13103941; doi:10.1158/2767-9764.CRC-25-0294)

Supplementary Figure S2: Souporcell results. For each sample, two UMAP plots are shown: UMAP derived from Seurat based on transcriptional data, colored by final integrated cell type (left) or by each sample's Souporcell clustering results (right). The K parameter for Souporcell clustering is also shown. K = 2 was used for all samples except those where a clear malignant cluster could not be distinguished. For DKO_2, DKO_3 and DKOAA_2, K was set to 3.

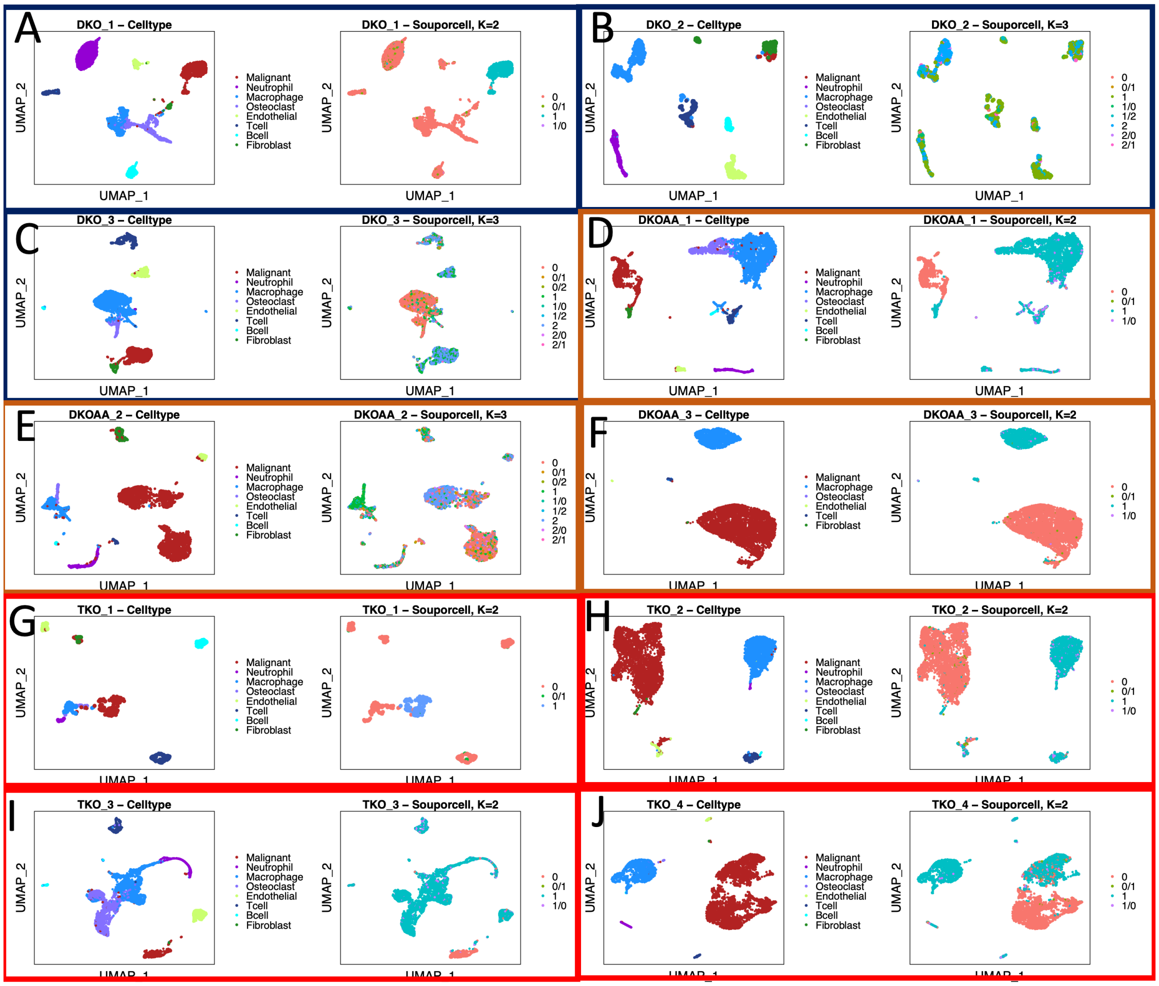

Supplement: Supplementary Figure S2 — Figure S2. Souporcell results. [file crc-25-0294_supplementary_figure_s2_suppsf2.pdf]
