# Supplementary material for: Single-Cell RNA Analysis of Murine Osteosarcoma Uncovers Skp2 Function in Metastasis, Genomic Instability, and Immune Activation and Reveals Additional Target Pathways
Source: Cancer Res Commun. 2026 Apr 23;6(4):923–45. doi: 10.1158/2767-9764.CRC-25-0294 (PMC13103941; doi:10.1158/2767-9764.CRC-25-0294)

Supplementary Figure S3: side by side comparison of InferCNV results for stromal cells (top) and malignant cells (bottom). Macrophages were used as reference celltypes in both. A: InferCNV results for stromal cells from all samples. B: InferCNV results for malignant cells from all samples.

A

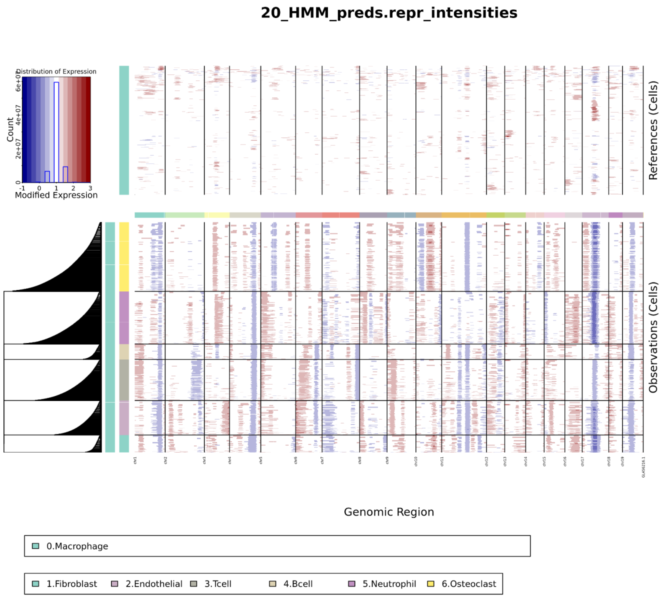

B

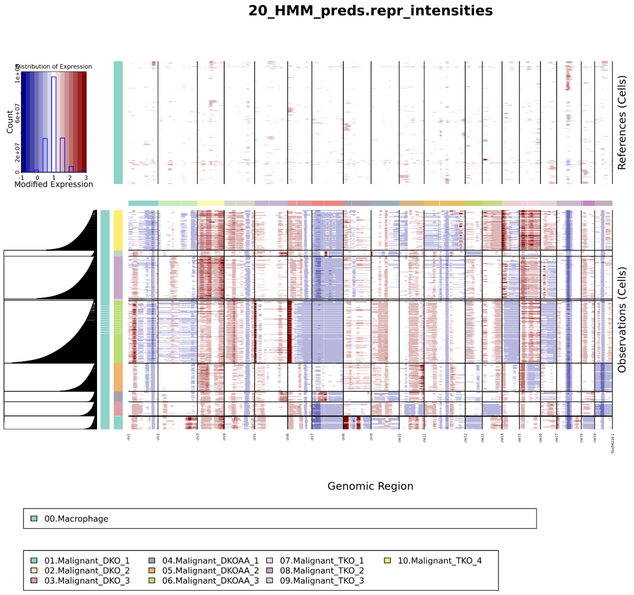

Supplement: Supplementary Figure S3 — Figure S3. side by side comparison of InferCNV results for stromal cells (top) and malignant cells (bottom). [file crc-25-0294_supplementary_figure_s3_suppsf3.pdf]
