# Supplementary material for: Single-Cell RNA Analysis of Murine Osteosarcoma Uncovers Skp2 Function in Metastasis, Genomic Instability, and Immune Activation and Reveals Additional Target Pathways
Source: Cancer Res Commun. 2026 Apr 23;6(4):923–45. doi: 10.1158/2767-9764.CRC-25-0294 (PMC13103941; doi:10.1158/2767-9764.CRC-25-0294)

Supplementary Figure S4. Leading edge genes from GSEA showing downregulation of invasive phenotypes in TKO and DKOAA relative to DKO. The intersects of leading edge genes from both TKO and DKOAA versus DKO enrichments are shown. A: Leading edge genes for Hallmark Epithelial-Mesenchymal Transition gene set. B: Leading edge genes for Reactome Extracellular Matrix Organization gene set. C: Leading edge genes for Gene Ontology Biological Process Cell Migration gene set.

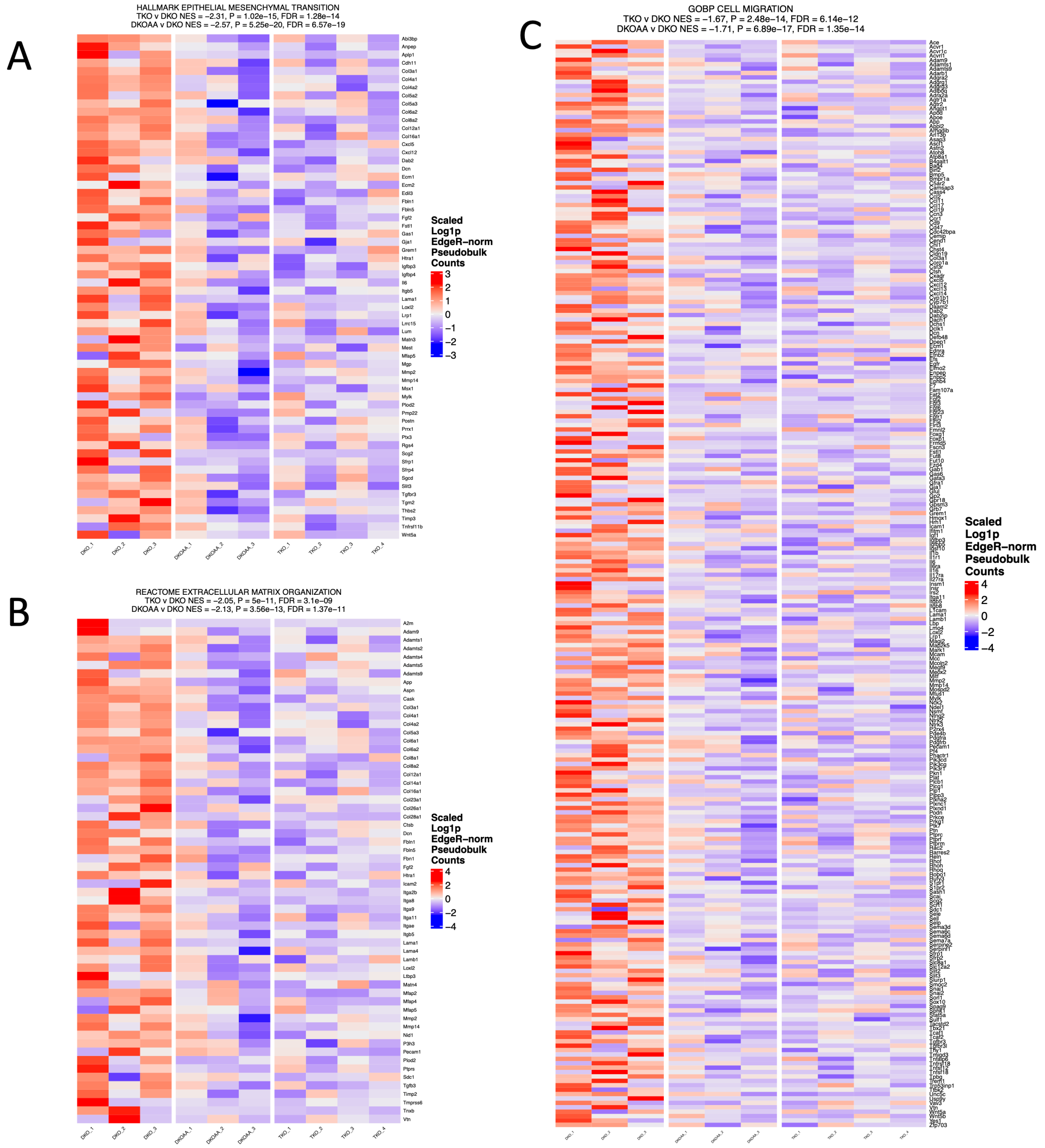

Supplement: Supplementary Figure S4 — Figure S4. Leading edge genes from GSEA showing downregulation of invasive phenotypes in TKO and DKOAA relative to DKO. [file crc-25-0294_supplementary_figure_s4_suppsf4.pdf]
