# Supplementary material for: Single-Cell RNA Analysis of Murine Osteosarcoma Uncovers Skp2 Function in Metastasis, Genomic Instability, and Immune Activation and Reveals Additional Target Pathways
Source: Cancer Res Commun. 2026 Apr 23;6(4):923–45. doi: 10.1158/2767-9764.CRC-25-0294 (PMC13103941; doi:10.1158/2767-9764.CRC-25-0294)

Supplementary Figure S5: Differential pathway gene set enrichment analysis among TKO vs DKO in all cell types, visualized as networks via aPEAR. Results are shown for each celltype.

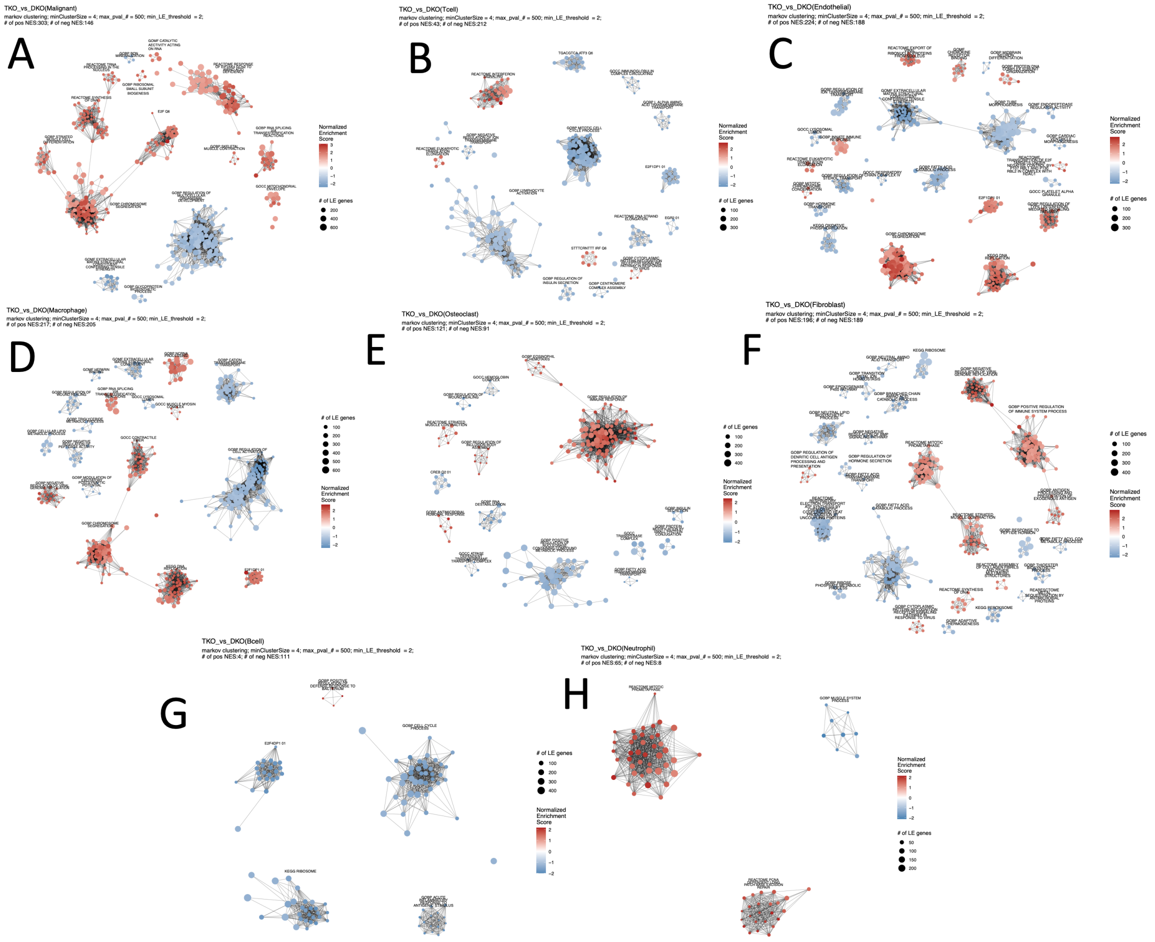

Supplement: Supplementary Figure S5 — Figure S5. Differential pathway gene set enrichment analysis among TKO vs DKO in all cell types, visualized as networks via aPEAR. [file crc-25-0294_supplementary_figure_s5_suppsf5.pdf]
