# Supplementary material for: Single-Cell RNA Analysis of Murine Osteosarcoma Uncovers Skp2 Function in Metastasis, Genomic Instability, and Immune Activation and Reveals Additional Target Pathways
Source: Cancer Res Commun. 2026 Apr 23;6(4):923–45. doi: 10.1158/2767-9764.CRC-25-0294 (PMC13103941; doi:10.1158/2767-9764.CRC-25-0294)

Supplementary Figure S7: GSEA of DKOAA and leading edge genes of interferon alpha and gamma response. A: Dot plot showing GSEA results of Hallmarks gene sets in DKOAA cell types relative to DKO. B-F: Heatmaps showing leading edge genes of Hallmark IFN-alpha and IFN-gamma response gene sets in the cell types indicated. The union of IFN-gamma and IFN-alpha leading edges is shown. Additionally, the union of TKO and DKOAA leading edges from these gene sets is shown for T cells and Osteoclasts, while for other celltypes, only the significant TKO enrichment's leading edge is shown.

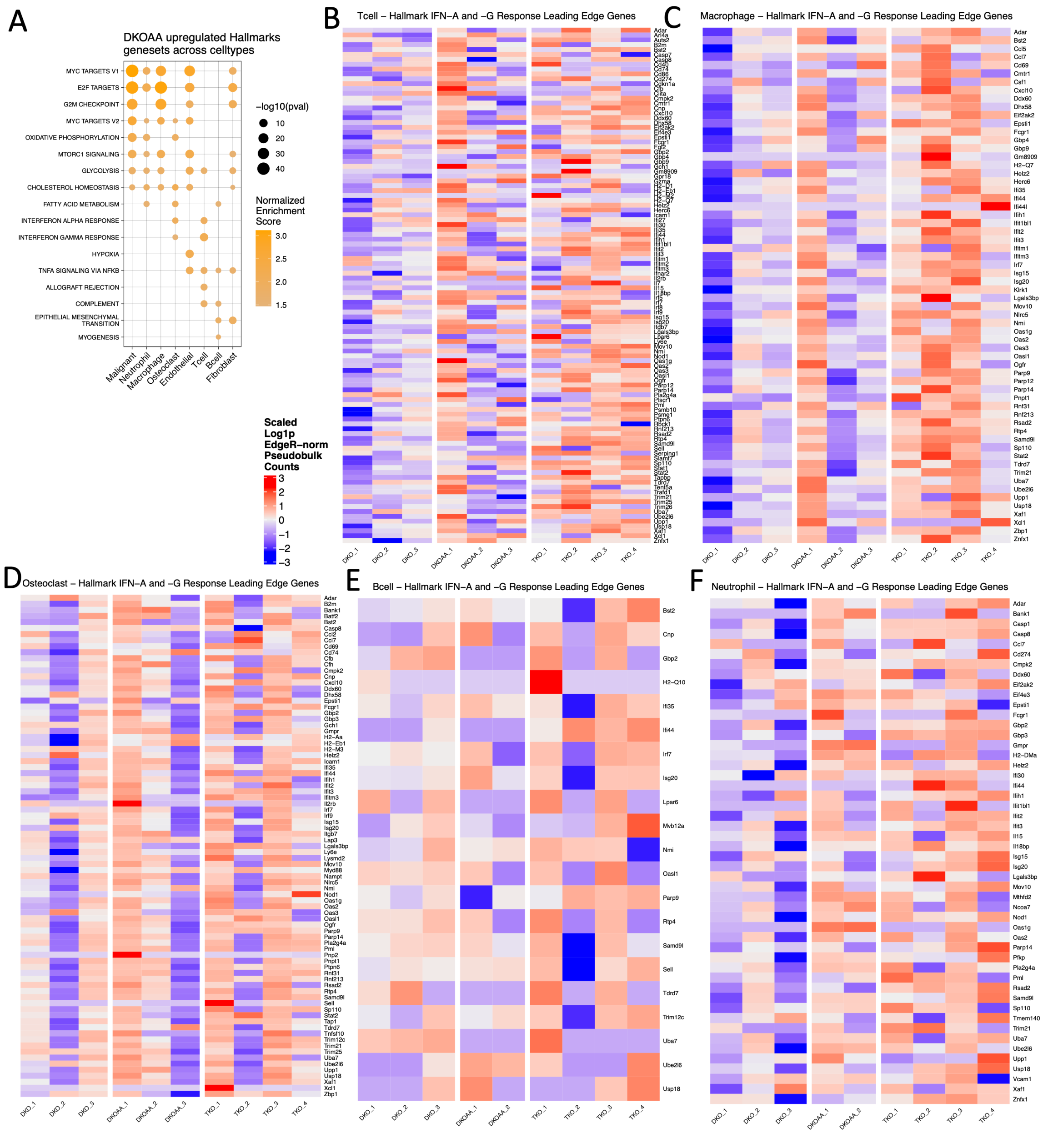

Supplement: Supplementary Figure S7 — Figure S7. GSEA of DKOAA and leading edge genes of interferon alpha and gamma response. [file crc-25-0294_supplementary_figure_s7_suppsf7.pdf]
