# Supplementary material for: Single-Cell RNA Analysis of Murine Osteosarcoma Uncovers Skp2 Function in Metastasis, Genomic Instability, and Immune Activation and Reveals Additional Target Pathways
Source: Cancer Res Commun. 2026 Apr 23;6(4):923–45. doi: 10.1158/2767-9764.CRC-25-0294 (PMC13103941; doi:10.1158/2767-9764.CRC-25-0294)

Supplementary Figure S8: GSEA of genesets related to cellular stress response. A,B: Dot plot showing GSEA results for gene sets significantly differentially expressed in TKO and DKOAA versus DKO, respectively. C – F: Heatmaps showing leading edge genes from significantly enriched gene sets of all cells. Star callouts are used to indicate that a significant enrichment was observed.

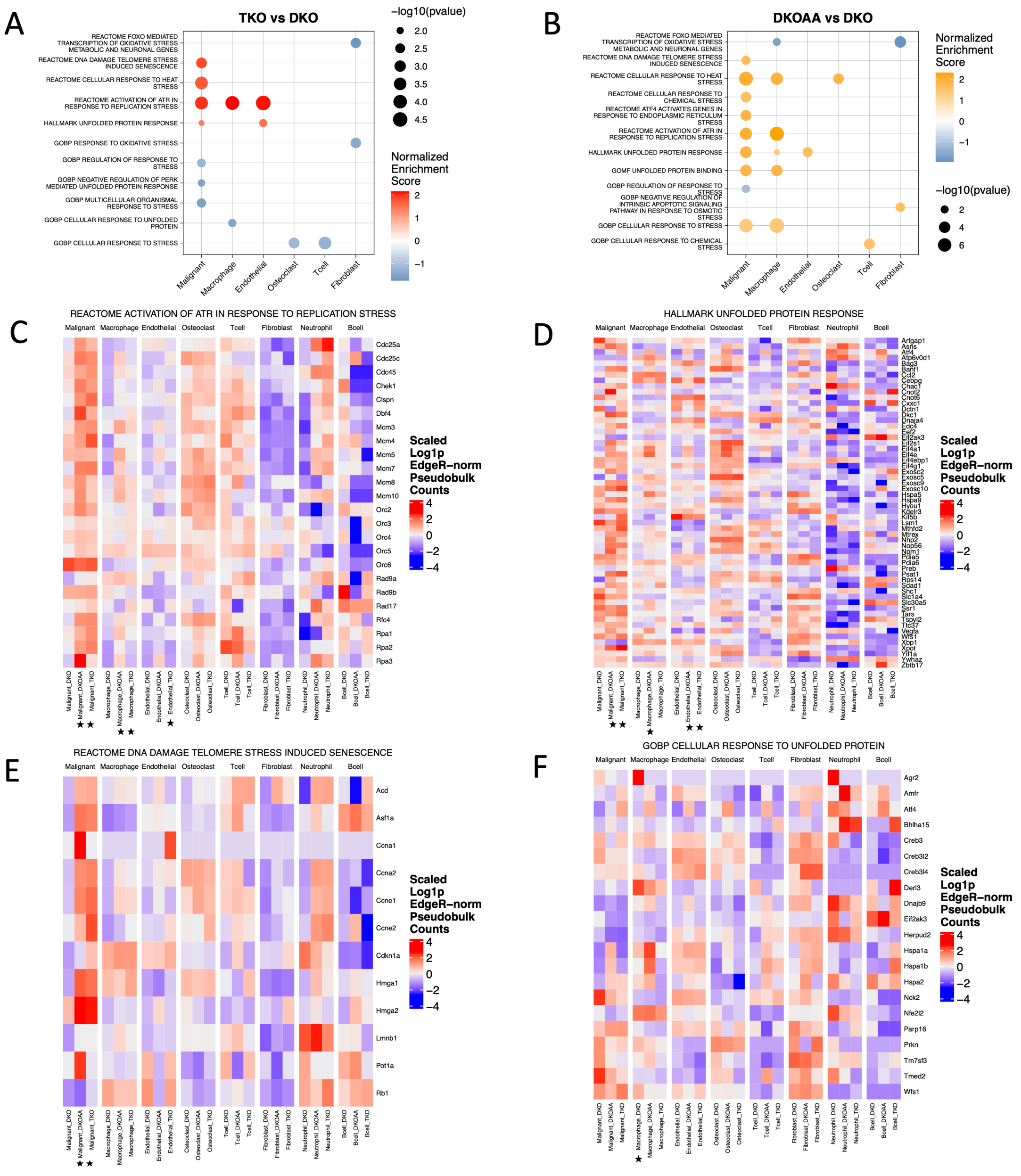

Supplement: Supplementary Figure S8 — Figure S8. GSEA of genesets related to cellular stress response. [file crc-25-0294_supplementary_figure_s8_suppsf8.pdf]
