# Supplementary material for: Single-Cell RNA Analysis of Murine Osteosarcoma Uncovers Skp2 Function in Metastasis, Genomic Instability, and Immune Activation and Reveals Additional Target Pathways
Source: Cancer Res Commun. 2026 Apr 23;6(4):923–45. doi: 10.1158/2767-9764.CRC-25-0294 (PMC13103941; doi:10.1158/2767-9764.CRC-25-0294)

Supplementary Figure S9. Mass spectrometry proteomics comparisons of TKO, DKOAA and DKO malignant tumor cell-derived cell lines. A: PCA of cell lines. Each sample is an individual cell line derived from a unique mice. DKO_1 was considered an outlier and not used in further analysis. B: Correlation of RNA with protein level expression in each model. C,D: Scatterplot showing each gene's protein-level and malignant cluster scRNAseq RNA-level differential expression result for TKO and DKO, respectively. Key SKP2-associated genes are highlighted in black, while genes associated with cell stress and antigen presentation are highlighted in red. E,F: Scatterplot with only significant DE genes at the protein and malignant cluster scRNAseq RNA-level.

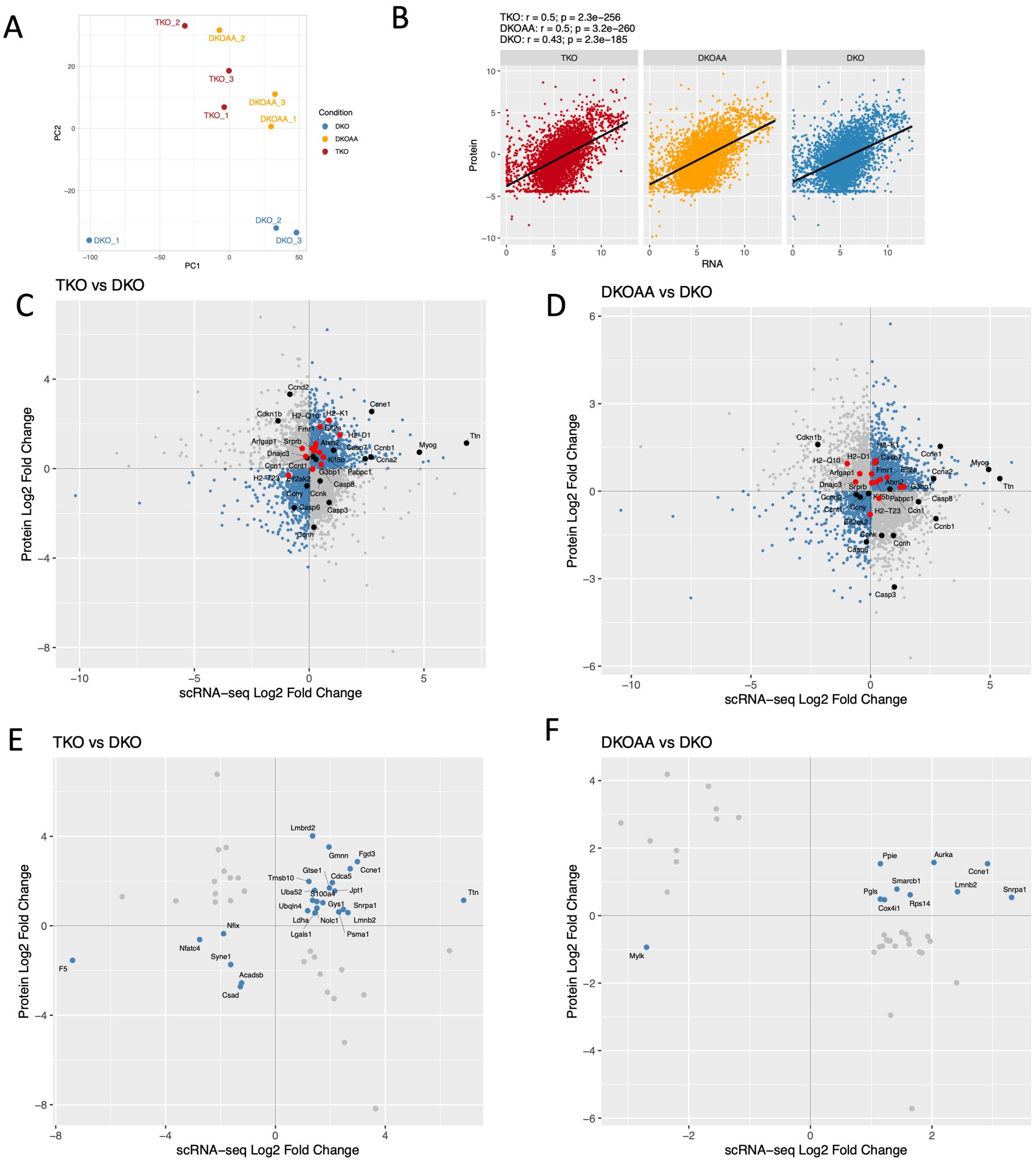

Supplement: Supplementary Figure S9 — Mass spectrometry proteomics comparisons of TKO, DKOAA and DKO malignant tumor cell-derived cell lines. [file crc-25-0294_supplementary_figure_s9_suppsf9.pdf]
