# Supplementary material for: Single-Cell RNA Analysis of Murine Osteosarcoma Uncovers Skp2 Function in Metastasis, Genomic Instability, and Immune Activation and Reveals Additional Target Pathways
Source: Cancer Res Commun. 2026 Apr 23;6(4):923–45. doi: 10.1158/2767-9764.CRC-25-0294 (PMC13103941; doi:10.1158/2767-9764.CRC-25-0294)

Supplementary Figure S11: CellChat analysis of cell signaling differences between OS tumors. A: Differential signaling strength among cell types in TKO vs DKO. Red = increased in TKO. B: Differential signaling strength among cell types in DKOAA vs DKO. C-E: Outgoing signaling pathways detected across TKO, DKOAA, and DKO. F-H: Incoming signaling pathways detected across TKO, DKOAA and DKO.

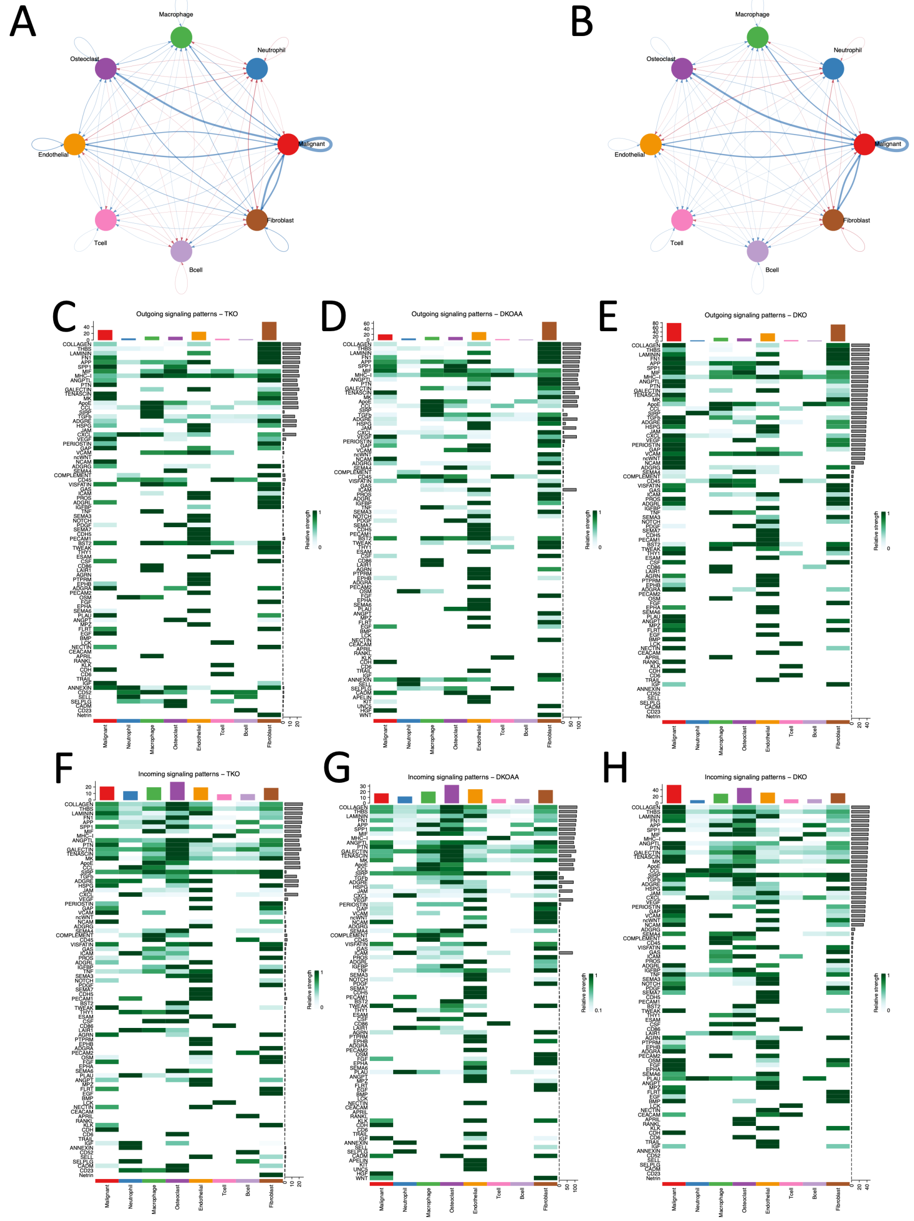

Supplement: Supplementary Figure S11 — Figure S11. CellChat analysis of cell signaling differences between OS tumors. [file crc-25-0294_supplementary_figure_s11_suppsf11.pdf]
