# Supplementary material for: Single-Cell RNA Analysis of Murine Osteosarcoma Uncovers Skp2 Function in Metastasis, Genomic Instability, and Immune Activation and Reveals Additional Target Pathways
Source: Cancer Res Commun. 2026 Apr 23;6(4):923–45. doi: 10.1158/2767-9764.CRC-25-0294 (PMC13103941; doi:10.1158/2767-9764.CRC-25-0294)

Supplementary Figure S12: Subclustering of individual cell types. Two UMAP plots are shown for each cell type: on the left, colored by Louvain sub-clusters; on the right, colored by OS models.

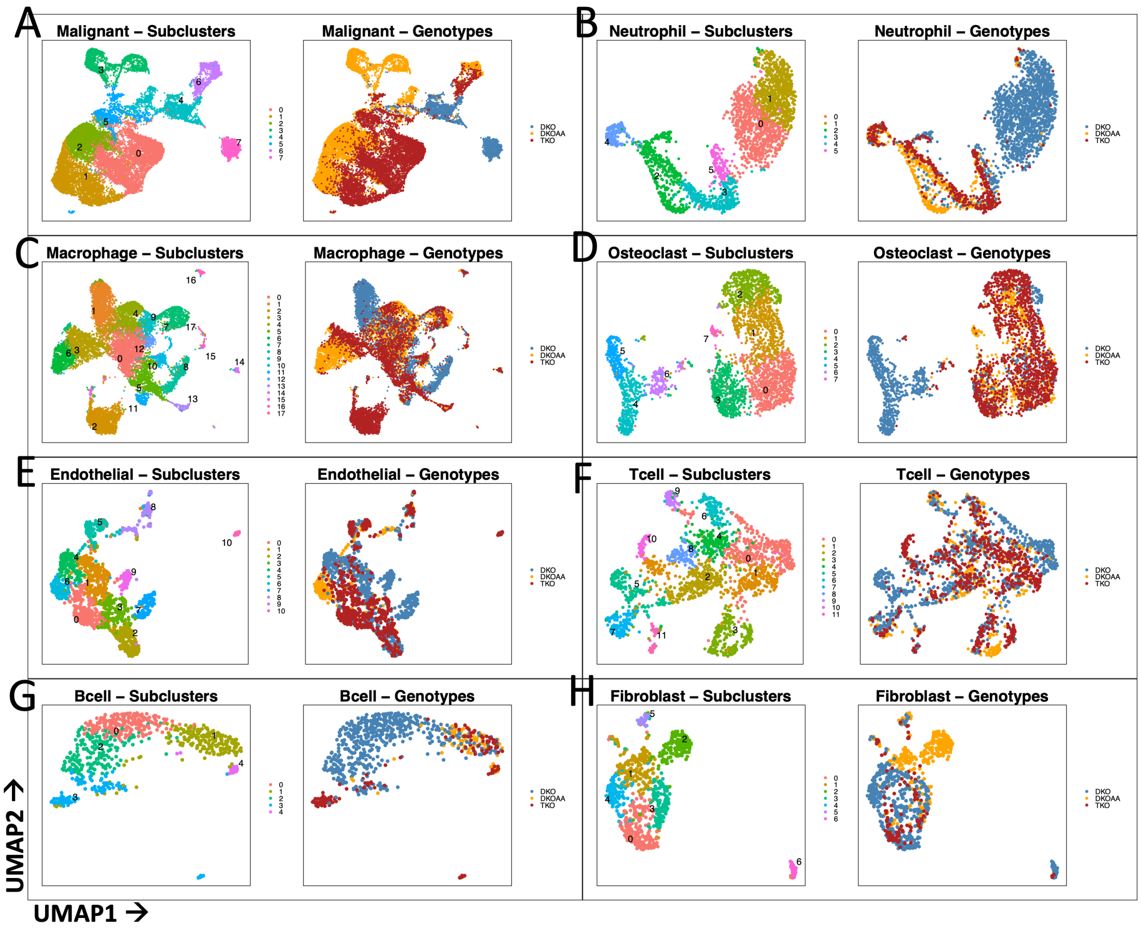

Supplement: Supplementary Figure S12 — Figure S12. Subclustering of individual cell types. [file crc-25-0294_supplementary_figure_s12_suppsf12.pdf]
