# Supplementary material for: Single-Cell RNA Analysis of Murine Osteosarcoma Uncovers Skp2 Function in Metastasis, Genomic Instability, and Immune Activation and Reveals Additional Target Pathways
Source: Cancer Res Commun. 2026 Apr 23;6(4):923–45. doi: 10.1158/2767-9764.CRC-25-0294 (PMC13103941; doi:10.1158/2767-9764.CRC-25-0294)

Supplementary Figure S13: Marker analysis for subclusters of individual cell types. The top 5 markers are shown for each sub-cluster.

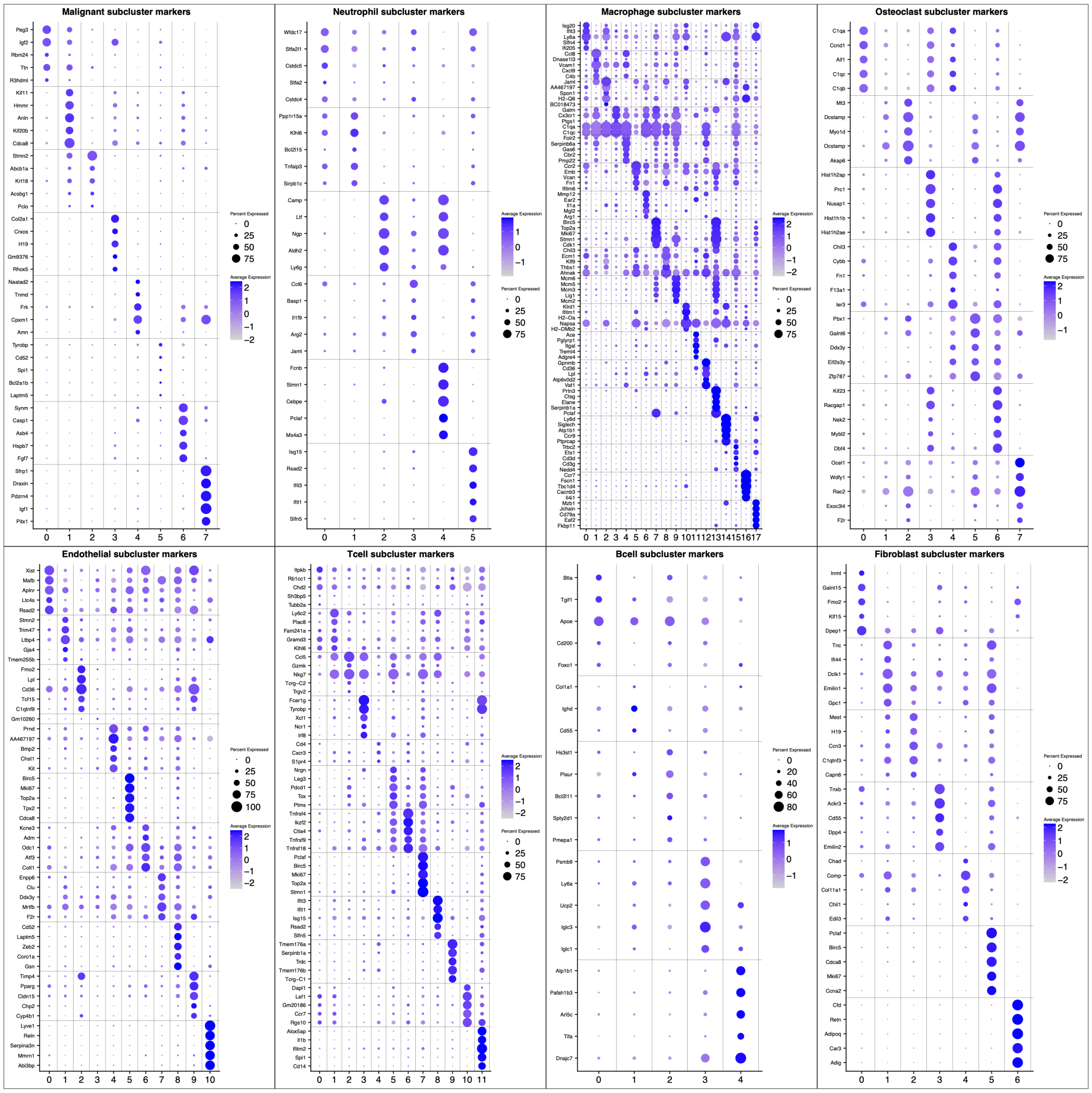

Supplement: Supplementary Figure S13 — Figure S13. Marker analysis for subclusters of individual cell types. [file crc-25-0294_supplementary_figure_s13_suppsf13.pdf]
