# Supplementary material for: Single-Cell RNA Analysis of Murine Osteosarcoma Uncovers Skp2 Function in Metastasis, Genomic Instability, and Immune Activation and Reveals Additional Target Pathways
Source: Cancer Res Commun. 2026 Apr 23;6(4):923–45. doi: 10.1158/2767-9764.CRC-25-0294 (PMC13103941; doi:10.1158/2767-9764.CRC-25-0294)

Supplementary Figure S14: Heatmaps showing proportions of subclusters for cell types containing at least one subcluster with a significant difference of proportion among OS models. Significant differences ($p < 0.05$) are denoted by cluster labels with asterisks. Propeller test of proportions from the Speckle package was used for statistical testing.

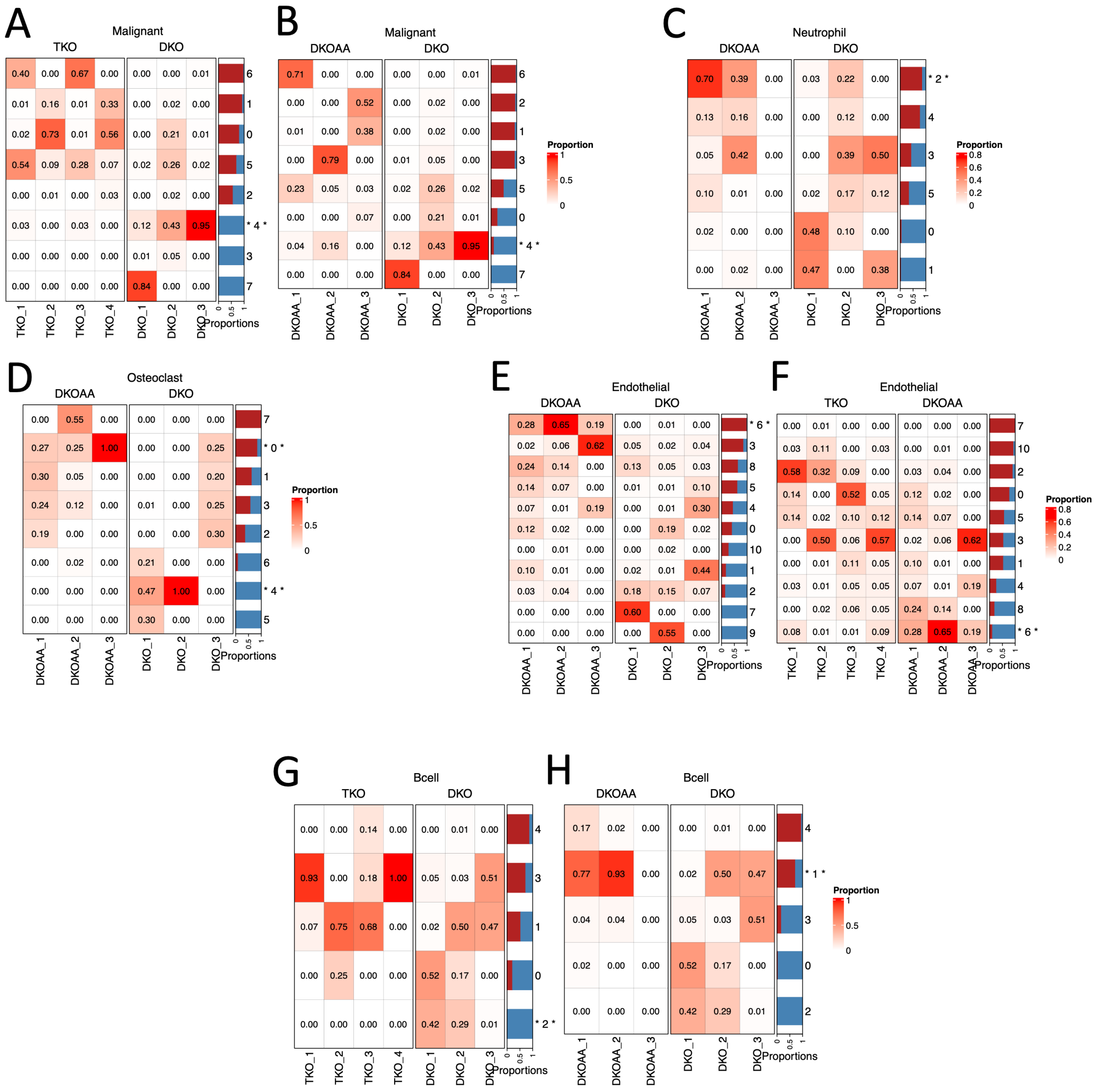

Supplement: Supplementary Figure S14 — Figure S14. Heatmaps showing proportions of subclusters for cell types containing at least one subcluster with a significant difference of proportion among OS models. [file crc-25-0294_supplementary_figure_s14_suppsf14.pdf]
