# Supplementary material for: Single-Cell RNA Analysis of Murine Osteosarcoma Uncovers Skp2 Function in Metastasis, Genomic Instability, and Immune Activation and Reveals Additional Target Pathways
Source: Cancer Res Commun. 2026 Apr 23;6(4):923–45. doi: 10.1158/2767-9764.CRC-25-0294 (PMC13103941; doi:10.1158/2767-9764.CRC-25-0294)

Supplementary Figure S15: E2f and Myc upregulation in TKO and DKOAA malignant cells. A: E2f and *Myc* family gene expression patterns across models for malignant cells. B: E2f and *Myc* family gene expression across samples for malignant cells. C: E2f and *Myc* transcriptional regulon scores computed by SCENIC. These are the only transcription factors from the genes shown in A and B for which SCENIC scores were available. D: UMAP of celltypes colored by cell cycle phase. E,F: Violin plots of S phase and G2/M phase scores, respectively. G-K: Heatmaps for the expression patterns of regulon genes from SCENIC for the transcription factors in panel C among malignant cells from TKO, DKOAA and DKO. L: Enrichment and leading-edge genes for the Reactome Apoptosis gene set among malignant cells from TKO, DKOAA and DKO. M: Violin plot of the *Myc* signature score calculated in all cells.

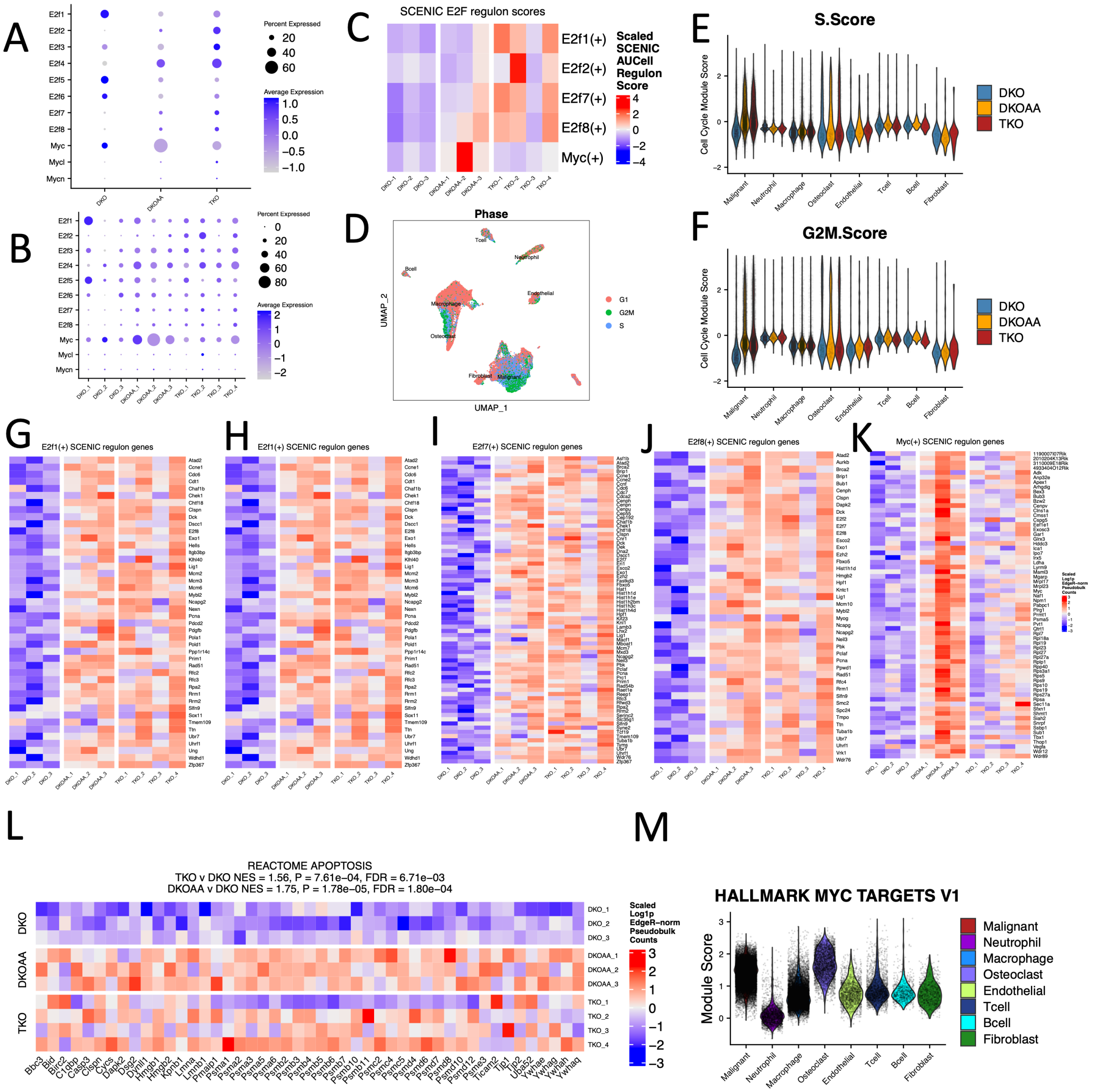

Supplement: Supplementary Figure S15 — Figure S15. E2f and Myc upregulation in TKO and DKOAA malignant cells. [file crc-25-0294_supplementary_figure_s15_suppsf15.pdf]
