# Supplementary material for: Single-Cell RNA Analysis of Murine Osteosarcoma Uncovers Skp2 Function in Metastasis, Genomic Instability, and Immune Activation and Reveals Additional Target Pathways
Source: Cancer Res Commun. 2026 Apr 23;6(4):923–45. doi: 10.1158/2767-9764.CRC-25-0294 (PMC13103941; doi:10.1158/2767-9764.CRC-25-0294)

Supplementary Figure S16: Clustering and markers of integrated human OS scRNA-seq data. A: Louvain clusters of integrated human scRNA-seq data (from RISC). B: Violin plot of number of genes affected by “extreme CNVs” (>=2x deletion or amplification). C: Canonical and data-driven markers plotted at the cluster level; the same markers as Fig 6D are shown. D: Data-driven markers for each cluster. E: Data-driven markers for each celltype after merging clusters of the same cell type.

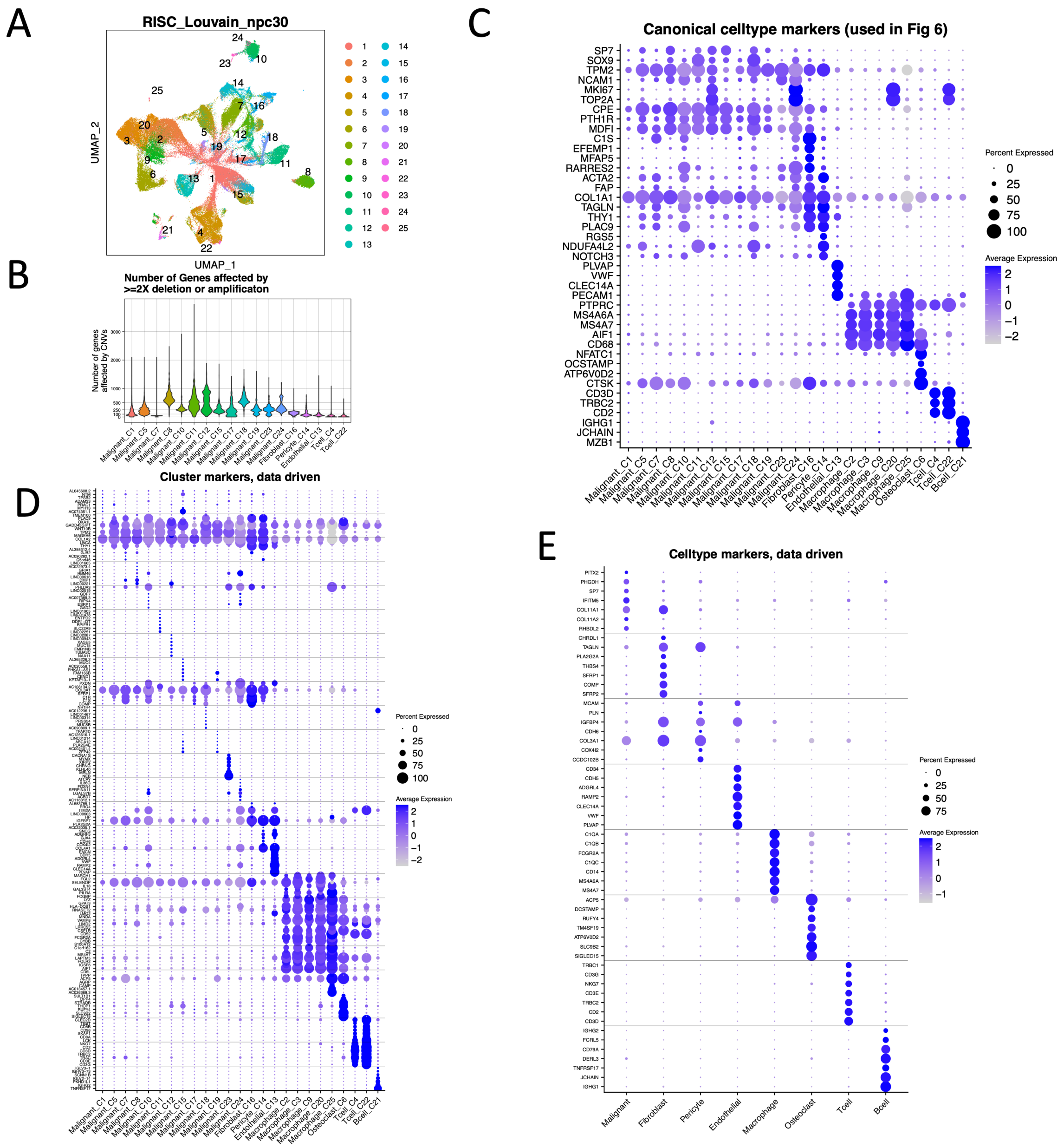

Supplement: Supplementary Figure S16 — Figure S16. Clustering and markers of integrated human OS scRNA-seq data [file crc-25-0294_supplementary_figure_s16_suppsf16.pdf]
