# Supplementary material for: Single-Cell RNA Analysis of Murine Osteosarcoma Uncovers Skp2 Function in Metastasis, Genomic Instability, and Immune Activation and Reveals Additional Target Pathways
Source: Cancer Res Commun. 2026 Apr 23;6(4):923–45. doi: 10.1158/2767-9764.CRC-25-0294 (PMC13103941; doi:10.1158/2767-9764.CRC-25-0294)

Supplementary Figure S17: Simplification of bone cell atlas annotations for usage in label transfer. A: UMAP of atlas data, colored by published clusters. B: UMAP colored by joined, simplified celltypes. C: Markers of simplified celltypes plotted in published clusters. D: Markers of simplified celltypes plotted after joining clusters to simplified, cell-type annotations.

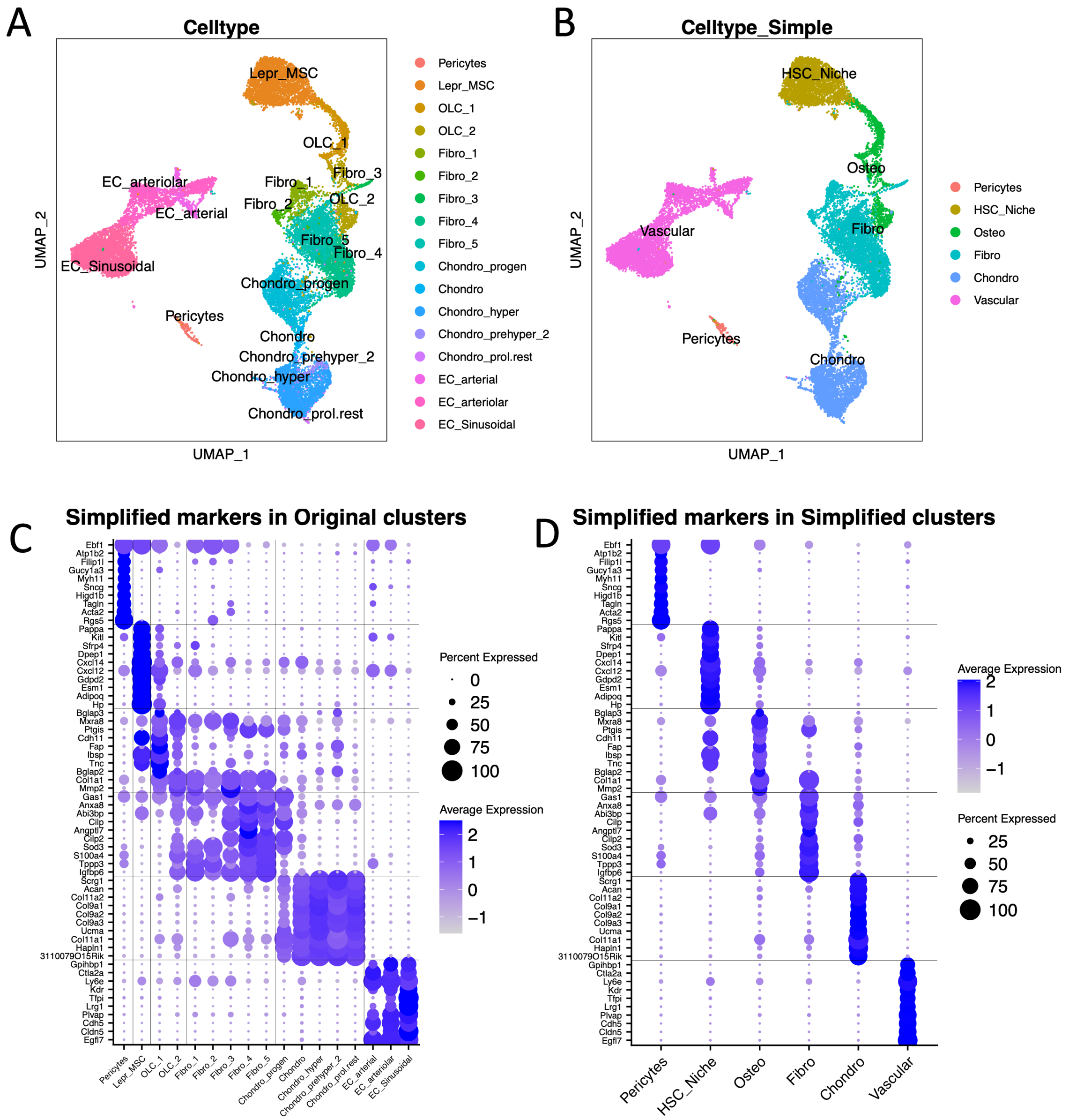

Supplement: Supplementary Figure S17 — Figure S17. Simplification of bone cell atlas annotations for usage in label transfer. [file crc-25-0294_supplementary_figure_s17_suppsf17.pdf]
