# Supplementary material for: Single-Cell RNA Analysis of Murine Osteosarcoma Uncovers Skp2 Function in Metastasis, Genomic Instability, and Immune Activation and Reveals Additional Target Pathways
Source: Cancer Res Commun. 2026 Apr 23;6(4):923–45. doi: 10.1158/2767-9764.CRC-25-0294 (PMC13103941; doi:10.1158/2767-9764.CRC-25-0294)

Supplementary Figure S18. Label transfer from bone atlas to non-malignant fibroblasts and endothelial cells. A, C: Label transfer results in OS non-malignant endothelial cells and non-malignant cancer associated fibroblasts, respectively. B,D: Label transfer scores in OS non-malignant endothelial cells and non-malignant cancer associated fibroblasts, respectively.

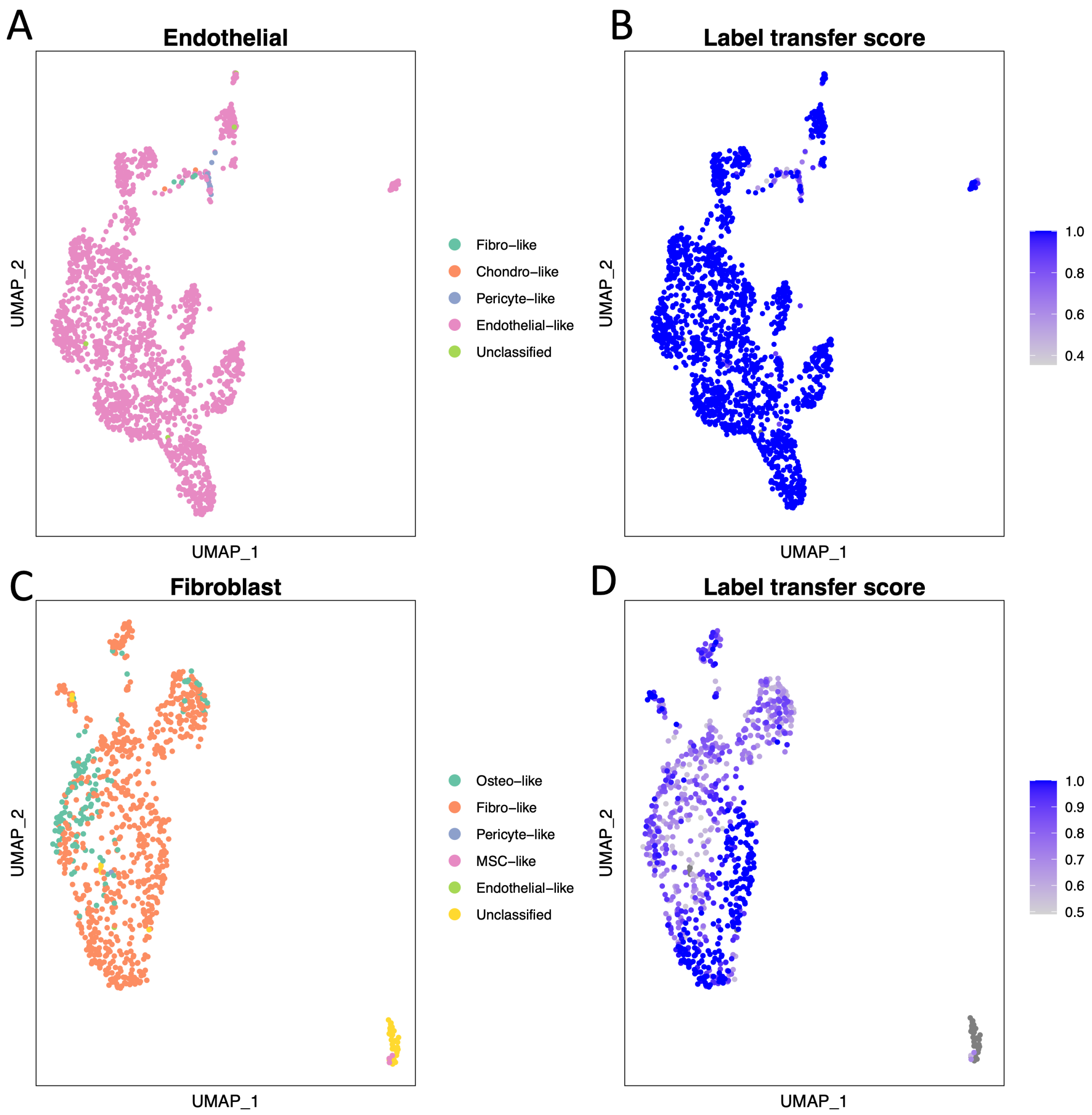

Supplement: Supplementary Figure S18 — Figure S18. Label transfer from bone atlas to non-malignant fibroblasts and endothelial cells. [file crc-25-0294_supplementary_figure_s18_suppsf18.pdf]
