# Supplementary material for: Single-Cell RNA Analysis of Murine Osteosarcoma Uncovers Skp2 Function in Metastasis, Genomic Instability, and Immune Activation and Reveals Additional Target Pathways
Source: Cancer Res Commun. 2026 Apr 23;6(4):923–45. doi: 10.1158/2767-9764.CRC-25-0294 (PMC13103941; doi:10.1158/2767-9764.CRC-25-0294)

Supplementary Figure S19. Markers and transcription factors from malignant cells stratified by pathologic subtype. A: Dotplot showing markers from the three most common inferred celltypes among malignant cells. B: SCENIC AUCell Regulon scores of transcription factors associated with the three most common inferred celltypes among malignant cells. C: *Asb5* expression among malignant subtypes and non-malignant cells. D: *Asb5* expression among malignant subtypes and non-malignant cells, stratified by OS models.

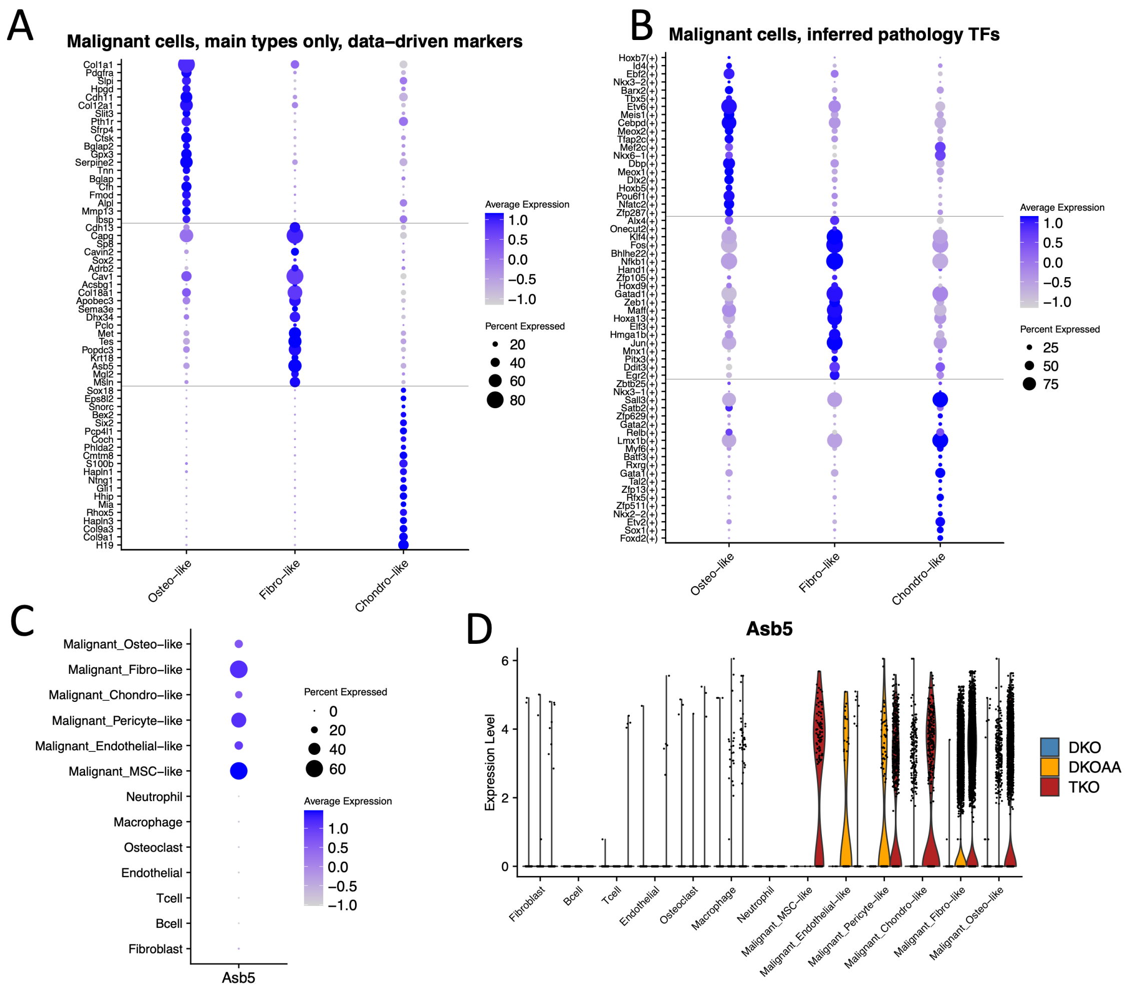

Supplement: Supplementary Figure S19 — Figure S19. Markers and transcription factors from malignant cells stratified by pathologic subtype. [file crc-25-0294_supplementary_figure_s19_suppsf19.pdf]
