# Supplementary material for: Single-Cell RNA Analysis of Murine Osteosarcoma Uncovers Skp2 Function in Metastasis, Genomic Instability, and Immune Activation and Reveals Additional Target Pathways
Source: Cancer Res Commun. 2026 Apr 23;6(4):923–45. doi: 10.1158/2767-9764.CRC-25-0294 (PMC13103941; doi:10.1158/2767-9764.CRC-25-0294)

Supplementary Figure S20. DE across OS models, stratified by pathologic subtype for which samples were available (including Osteo and Fibro-like, but not chondro-like). A, C: TKO vs DKO differentially expressed Hallmarks gene sets in Osteo- and Fibro-like malignant cells, respectively. B,D: DKOAA vs DKO differentially expressed Hallmarks gene sets in Osteo- and Fibro-like malignant cells, respectively.

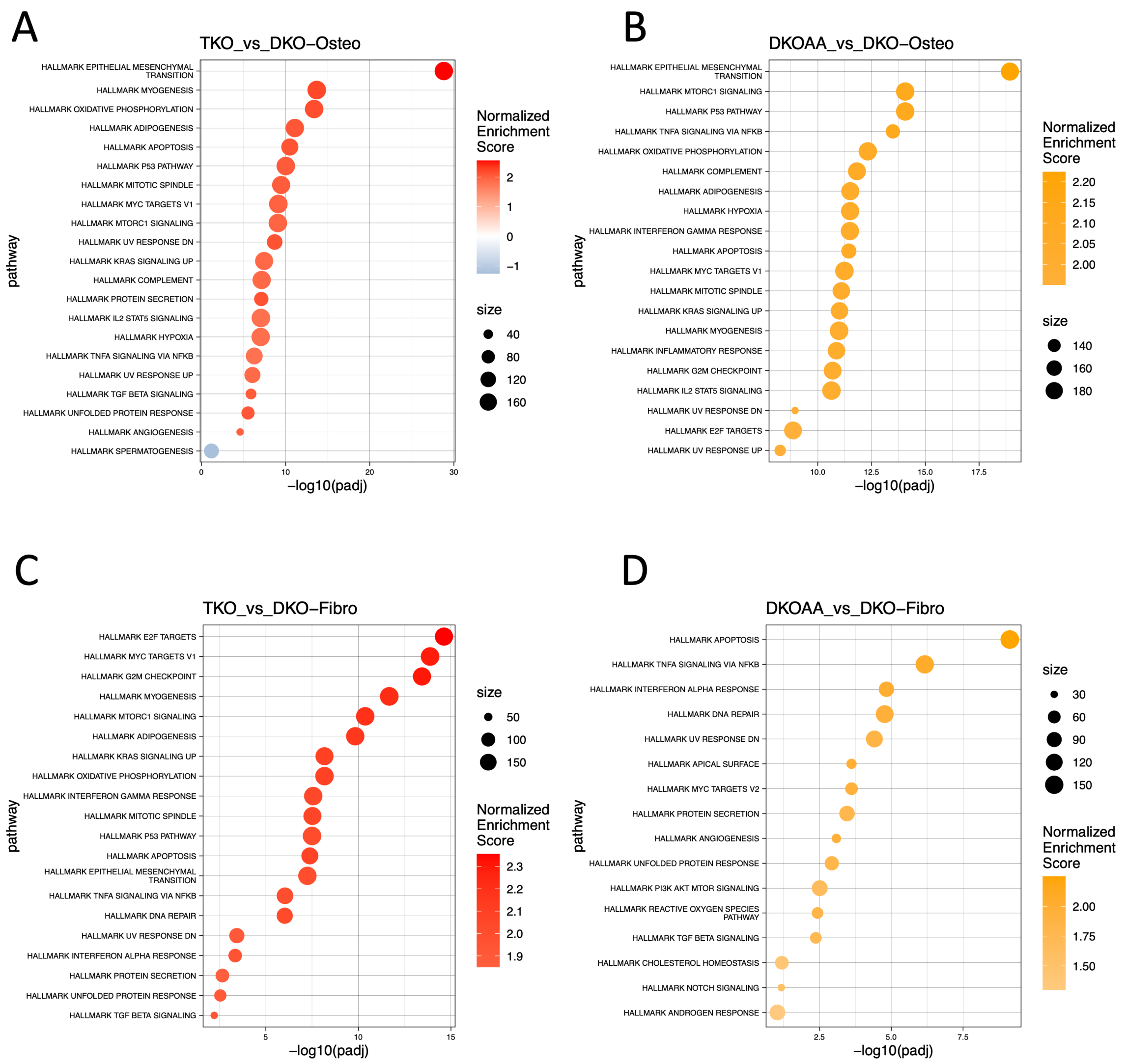

Supplement: Supplementary Figure S20 — Figure S20. DE across OS models, stratified by pathologic subtype for which samples were available (including Osteo and Fibro-like, but not chondro-like). [file crc-25-0294_supplementary_figure_s20_suppsf20.pdf]
