# Supplementary material for: Single-Cell RNA Analysis of Murine Osteosarcoma Uncovers Skp2 Function in Metastasis, Genomic Instability, and Immune Activation and Reveals Additional Target Pathways
Source: Cancer Res Commun. 2026 Apr 23;6(4):923–45. doi: 10.1158/2767-9764.CRC-25-0294 (PMC13103941; doi:10.1158/2767-9764.CRC-25-0294)

Supplementary Figure S21. EMT gene expression in Osteo vs non-Osteo samples. A: EMT related genes in all samples. B: EMT related genes in osteo-samples, averaged for samples in each of the three OS models. C: EMT related genes in non-osteo samples (fibro and chondro), averaged for samples in each of the three OS models.

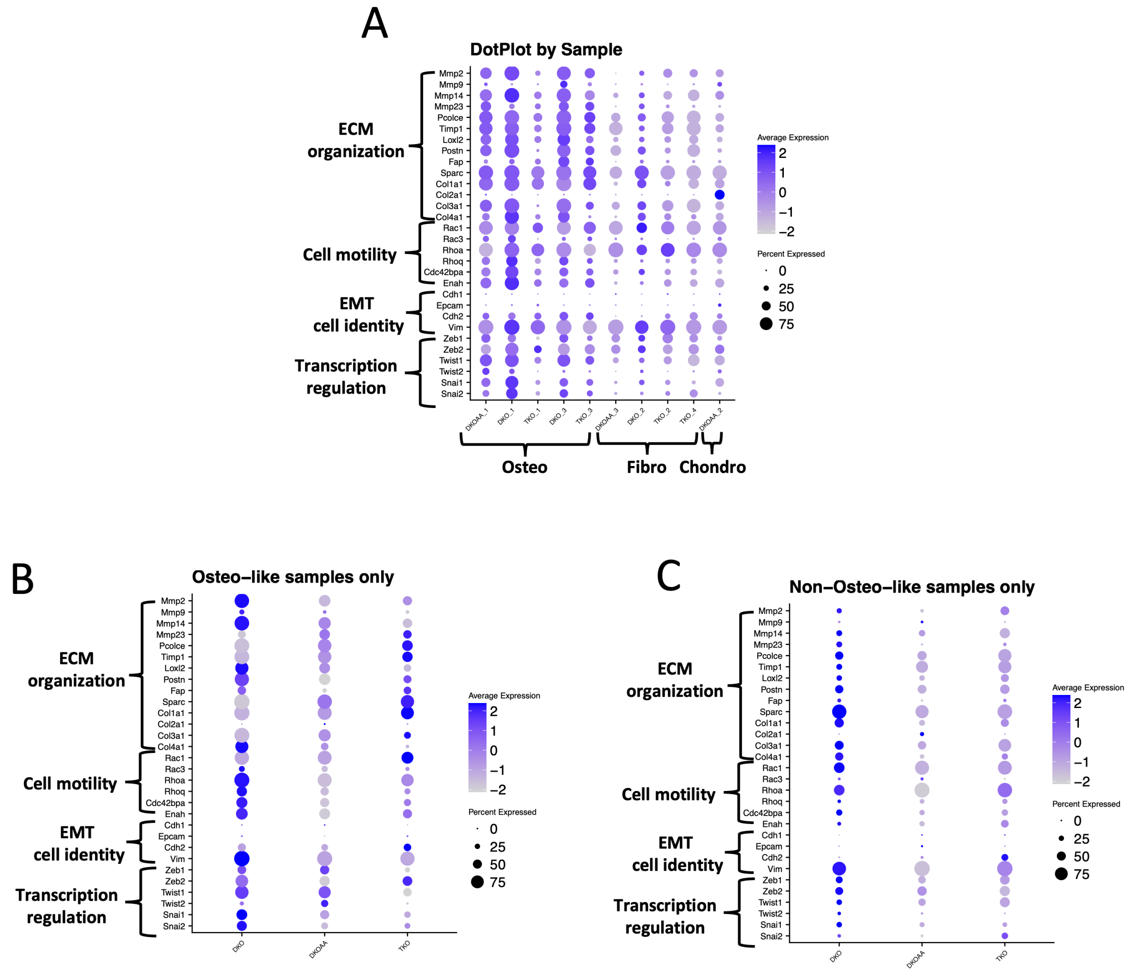

Supplement: Supplementary Figure S21 — Figure S21. EMT gene expression in Osteo vs non-Osteo samples. [file crc-25-0294_supplementary_figure_s21_suppsf21.pdf]
