# Supplementary material for: Single-Cell RNA Analysis of Murine Osteosarcoma Uncovers Skp2 Function in Metastasis, Genomic Instability, and Immune Activation and Reveals Additional Target Pathways
Source: Cancer Res Commun. 2026 Apr 23;6(4):923–45. doi: 10.1158/2767-9764.CRC-25-0294 (PMC13103941; doi:10.1158/2767-9764.CRC-25-0294)
Supplement: Supplementary Figure S22 — Figure S22. Expression of TKO myogenesis program in human OS tumors. [file crc-25-0294_supplementary_figure_s22_suppsf22.pdf]

Supplementary Figure S22. Expression of TKO myogenesis program in human OS tumors. A: Dotplot of relevant genes, including *SKP2*, p57 (*CDKN1C*), *FBXL13*, and *ASB5*; Myogenic TFs; and the TKO upregulated Myogenesis signature. B: Featureplot showing expression score of the TKO upregulated myogenesis signature. The genes include the bottom genes of the dotplot as well as *MYOG*, the same as the signature shown in Fig 8G. C: Table showing cell numbers in clusters and patient samples. Star callout indicates the malignant cluster positive for myogenesis signal, and the associated patient sample which contributed most cells to that cluster.

A

B

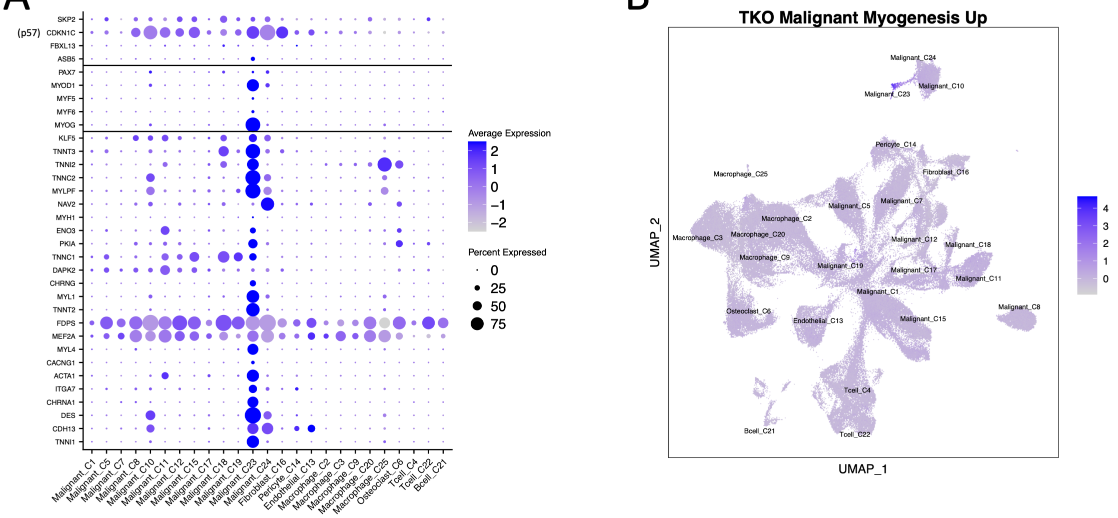

C

Clusters / Samples

Clusters	FrontOncol1_GSM4952363	FrontOncol2_GSM4952364	FrontOncol3_GSM4952365	FrontOncol4_GSM5155198	FrontOncol5_GSM5155199	FrontOncol6_GSM5155200	NatComm1_BC2	NatComm2_BC3	NatComm3_BC5	NatComm4_BC6	NatComm5_BC10	NatComm6_BC11	NatComm7_BC16	★ NatComm8_BC17	NatComm9_BC20	NatComm10_BC21	NatComm11_BC22
Malignant_C1	1048	417	203	60	55	351	361	980	1589	763	7246	7340	1424	450	346	1739	1445
Malignant_C5	25	108	155	15	7	39	58	1904	6784	40	2765	225	129	13	5	24	73
Malignant_C7	7	60	109	3	3	101	105	21	317	8095	178	10	11	13	26	72	16
Malignant_C8	793	1556	5048	902	58	87	11	22	51	0	3	19	59	20	15	19	9
Malignant_C10	0	0	0	0	0	0	0	0	0	0	0	0	0	5866	0	1	9
Malignant_C11	14	76	15	119	11	147	12	9	14	0	16	106	16	77	0	4680	284
Malignant_C12	55	215	310	104	63	1822	13	482	1280	24	443	256	65	13	8	79	27
Malignant_C15	1	5	3	4	4	9	20	12	10	0	7	2519	1122	1	0	10	16
Malignant_C17	1	15	13	7	3	43	2	8	4	1	0	16	112	495	382	592	364
Malignant_C18	0	2	0	0	6	3	0	1	1	0	2	9	6	22	1	2	2009
Malignant_C19	90	77	4	4	11	9	1	0	2	0	250	23	559	2	2	12	8
★ Malignant_C23	6	0	1	1	0	0	0	0	4	0	0	0	0	324	0	0	37
Malignant_C24	0	0	0	0	0	0	0	0	0	0	0	0	0	242	0	0	0
Fibroblast_C16	7	9	40	50	0	292	0	0	175	3	298	15	3	59	630	6	896
Pericyte_C14	24	125	253	69	49	89	1552	157	409	167	143	29	355	38	63	797	59
Endothelial_C13	38	232	184	78	141	143	674	273	721	387	267	1	407	218	370	1	285
Macrophage_C2	139	272	63	42	159	51	1133	3776	3378	2842	305	223	1904	63	44	37	142
Macrophage_C3	2909	1637	47	785	5334	250	23	46	12	84	2	10	595	796	342	474	593
Macrophage_C9	309	307	53	752	294	797	519	168	169	630	406	80	623	284	356	70	453
Macrophage_C20	280	48	0	16	180	7	1	2	0	13	1	1	101	60	13	23	28
Macrophage_C25	0	0	0	0	0	0	0	0	0	0	0	0	0	143	0	0	0
Osteoclast_C6	1118	844	484	135	112	38	250	1233	722	2062	148	0	582	124	1631	0	1
Tcell_C4	287	689	68	462	1418	2667	217	3120	50	95	527	163	885	439	1114	0	781
Tcell_C22	17	29	2	6	225	103	0	37	0	0	0	1	22	15	1	1	8
Bcell_C21	1	2	0	2	1	703	0	0	0	0	0	3	1	1	3	0	3
