# Supplementary material for: Single-Cell RNA Analysis of Murine Osteosarcoma Uncovers Skp2 Function in Metastasis, Genomic Instability, and Immune Activation and Reveals Additional Target Pathways
Source: Cancer Res Commun. 2026 Apr 23;6(4):923–45. doi: 10.1158/2767-9764.CRC-25-0294 (PMC13103941; doi:10.1158/2767-9764.CRC-25-0294)

Supplementary Figure S23. Working model summarizing main findings from mouse OS models. *Skp2* functional disruption led to differential expression of both anti-cancer and pro-cancer pathways, representing a battle between different forces in malignant cells and TMEs, which eventually result in delayed but not complete blockage of tumor development. Even the tumors escaped from *Skp2* disruption may experience the anti-cancer benefit of *Skp2* reduction, leading to slow and less aggressive tumors. Figure was created with BioRender.

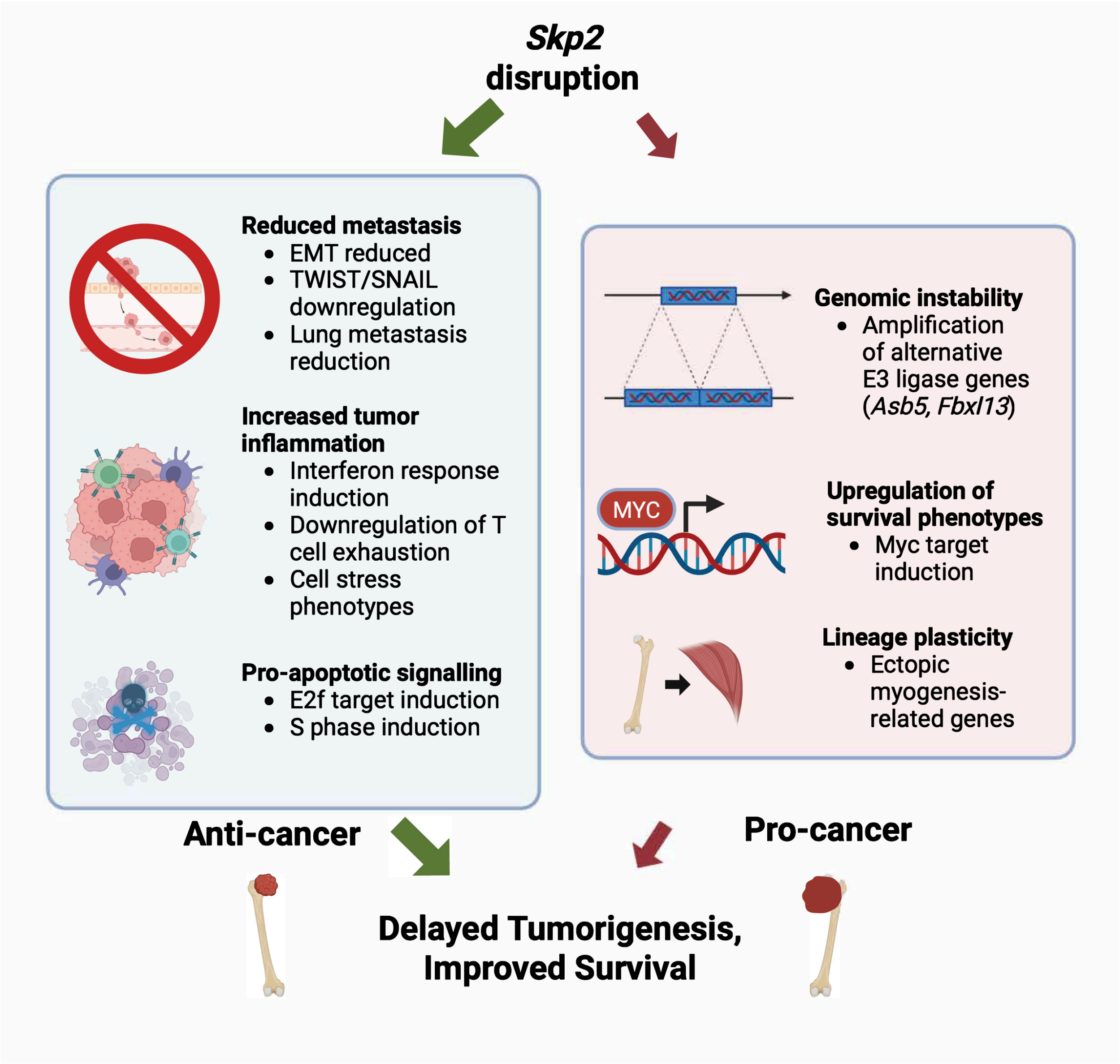

Supplement: Supplementary Figure S23 — Figure S23. Working model summarizing main findings from mouse OS models. [file crc-25-0294_supplementary_figure_s23_suppsf23.pdf]
