# Supplementary material for: Single-Cell RNA Analysis of Murine Osteosarcoma Uncovers Skp2 Function in Metastasis, Genomic Instability, and Immune Activation and Reveals Additional Target Pathways
Source: Cancer Res Commun. 2026 Apr 23;6(4):923–45. doi: 10.1158/2767-9764.CRC-25-0294 (PMC13103941; doi:10.1158/2767-9764.CRC-25-0294)

Supplementary Figure S10: Analysis of Interferon related genes. A: UMAP plots showing expression patterns of interferon genes. B,C: Dotplot and heatmap showing expression differences of interferon related genes across OS models, stratified by celltype.

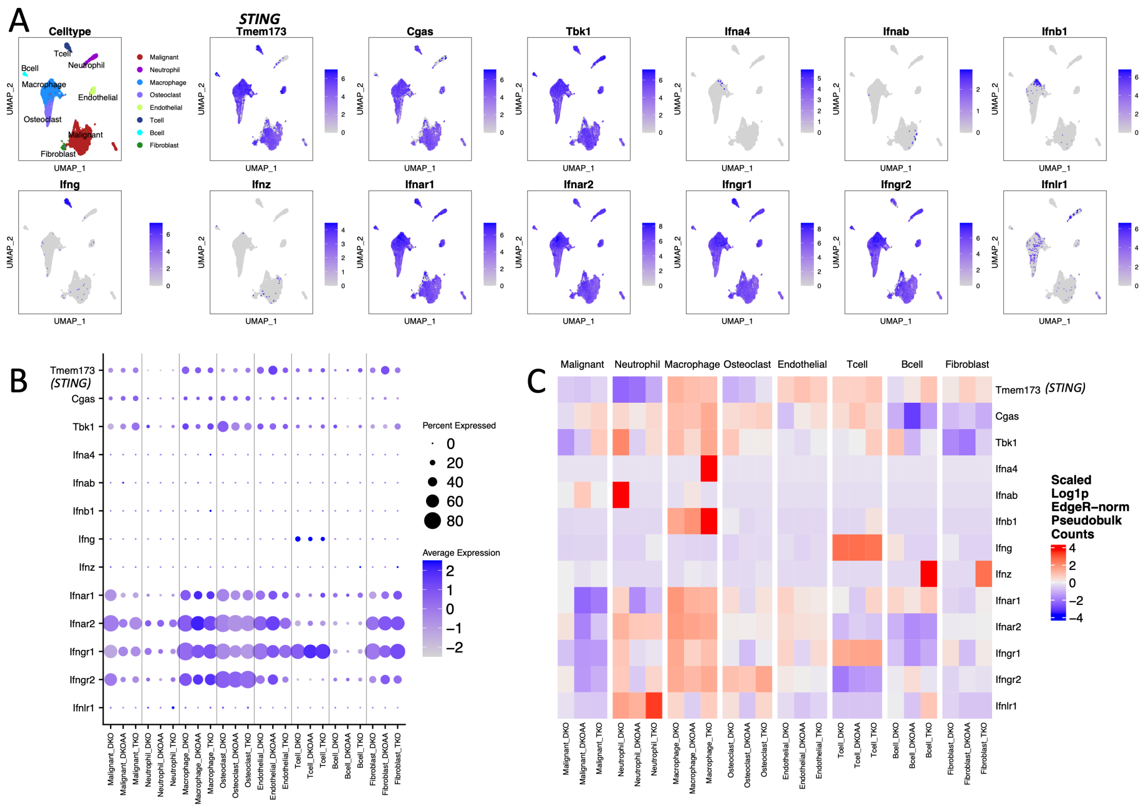

Supplement: Supplementary Figure S10 — Figure S10. Analysis of Interferon related genes. [file crc-25-0294_supplementary_figure_s10_suppsf10.pdf]
